# Novel Role of Gut‐Derived *Roseburia Intestinalis* in Safeguarding Intestinal Barrier Integrity and Microenvironment Homeostasis During Arsenic Exposure

**DOI:** 10.1002/advs.202511895

**Published:** 2025-08-19

**Authors:** Lixiao Zhou, Chunsong Wang, Jieying Gao, Xu Wu, Geng Li, Xuejun Jiang, Yinyin Xia, Jun Zhang, Bo Lv, Feng Zhao, Hongyang Zhang, Huifeng Pi, Jingfu Qiu, Shangcheng Xu, Zhen Zou, Chengzhi Chen

**Affiliations:** ^1^ Department of Occupational and Environmental Health School of Public Health Chongqing Medical University Chongqing 400016 People's Republic of China; ^2^ Research Center for Environment and Human Health Chongqing Medical University Chongqing 400016 People's Republic of China; ^3^ Department of Health Laboratory Technology School of Public Health Chongqing Medical University Chongqing 400016 People's Republic of China; ^4^ Center of Experimental Teaching for Public Health Experimental Teaching and Management Center Chongqing Medical University Chongqing 400016 People's Republic of China; ^5^ Molecular Biology Laboratory of Respiratory Disease Institute of Life Sciences Chongqing Medical University Chongqing 400016 People's Republic of China; ^6^ Department of Occupational Health Army Medical University Chongqing 400037 People's Republic of China; ^7^ Chongqing Key Laboratory of Prevention and Treatment for Occupational Diseases and Poisoning Chongqing 400060 China; ^8^ National Emergency Response Team for Sudden Poisoning The First Affiliated Hospital of Chongqing Medical and Pharmaceutical College Chongqing 400060 China

**Keywords:** arsenic, gut microbiota, intestinal barriers, *Roseburia intestinalis*

## Abstract

As a well‐known metalloid, arsenic usually causes human intestinal disorders via contaminated drinking water. However, the mechanisms underlying how arsenic induces intestinal injury remain unresolved, and the effective means of intervention are very limited. By establishing an acute arsenic exposure animal model, this work shows that arsenic disrupts the mechanical, chemical, immunological, and biological barriers of the intestine, and thereby changes the microenvironment in the gut. We further verify that the administration of fecal microbiota transplantation with a healthy gut microbiome alleviates the intestinal damage induced by arsenic. Intriguingly, by using 16S rRNA sequencing and anaerobic culture, we identify a novel role of gut‐derived strain, *Roseburia intestinalis*, which exhibits significant protection against arsenic‐induced intestinal toxicity in mice. By applying non‐targeted metabolomics after arsenic exposure, this work further establishes the beneficial effects and the potential metabolites associated with *Roseburia intestinalis*, including cacodylic acid, carindone, 3‐hydroxymelatonin and l‐galacto‐2‐heptulose, etc. Transcriptomic analysis reveals that the protective effects of *Roseburia intestinalis* against arsenic‐induced intestinal injury include mainly immune‐related pathways. Taken together, these findings highlight that supplementation with gut‐derived *Roseburia intestinalis* is an alternative strategy that could be used in the prevention and treatment of arsenic‐related intestinal disorders.

## Graphical abstract

Arsenic exposure disrupts intestinal barriers and gut microenvironment. Fecal microbiota transplantation (FMT) alleviates arsenic‐induced damage, with gut‐derived *Roseburia intestinalis* (R.i) identified as a key protective strain. R.i administration counters arsenic toxicity through immunomodulatory pathways and metabolites. Supplementation with R.i presents a novel therapeutic strategy against arsenic‐related intestinal disorders.

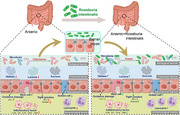

## 1 Introduction

Arsenic is a classic metalloid that has been found in more than 300 minerals on Earth. The arsenic ores dissolve and are released into the soil and water by natural weathering, oxidation, and erosion of sulfide minerals,^[^ ^1^ ^]^ thus leading to a widespread contamination. Although the World Health Organization (WHO) has set the allowed arsenic concentration in drinking water at a level below 0.01 mg L^−1^, the concentration of arsenic can reach 0.15–4.4 mg L^−1^ in the natural environment in some Asian countries, such as Bangladesh, India, and China.^[^ ^2^ ^]^ Humans are generally exposed to inorganic arsenic, such as mono‐ and dianion forms of arsenic acids, through arsenic‐contaminated water or the diet in a chronic manner.^[^ ^3^ ^]^ Notably, in addition to chronic exposure, acute or subacute arsenic poisoning, which usually occurs in cases of drug abuse or arsenic contaminated drinking water poisoning, can't be ignored.^[^ ^4^, ^5^ ^]^ A large body of evidence has indicated that the ingested arsenic can accumulate in nearly all tissues, such as the intestine, brain, lung, liver, kidney, bladder, skin, and even the bone marrow,^[^ ^6^ ^]^ thereby resulting in various disorders, including gastrointestinal disease, cardiovascular diseases, neurological disorders, and multiple types of cancer.^[^ ^7^, ^8^, ^9^, ^10^ ^]^

After ingestion, approximately 80% of arsenic is absorbed by the gastrointestinal tract.^[^ ^11^ ^]^ As the first line of defense against ingested xenobiotics, the small intestine is a primary organ essential for nutrient digestion and absorption, which relies on its normal morphology and intestinal barrier function to carry out its roles.^[^ ^12^ ^]^ Over the past few decades, accumulating evidence has suggested that arsenic has a detrimental impact on the intestinal function. Clinical symptoms such as dyspepsia and gastroenteritis have been found in arsenic‐exposed populations.^[^ ^13^, ^14^ ^]^ Laboratory studies have shown that exposure to arsenic significantly increases intestinal permeability and disturbs intestinal functions.^[^ ^15^ ^]^ Morphological analysis revealed that arsenic induced a thinner muscular layer in the intestine, shortened the intestinal villi and even ruptured the intestinal villi.^[^ ^16^ ^]^ To explain the adverse effects of arsenic, numerous mechanisms have been proposed, including the inhibition of intestinal epithelial tight junction proteins, redox imbalance, metabolic distress, inflammation, and immune disruption.^[^ ^17^, ^18^, ^19^, ^20^, ^21^ ^]^ Although these studies have made considerable progress in revealing the genes and pathways involved in intestinal damage caused by arsenic, the detailed mechanisms remain largely unclear, and there is a lack of effective targets for the prevention and treatment of such damage.

The gut microbiota plays an essential role in maintaining intestinal homeostasis. Disruption of the balance of the microbial population and function provokes disruption of the intestinal barrier and induces aberrant immune responses, which ultimately contribute to intestinal dysfunction.^[^ ^22^ ^]^ An increasing number of studies have shown that there is a reciprocal and bidirectional interaction between arsenic exposure and the gut microbiota. Arsenic intake significantly alters the composition and metabolic landscape of the gut microbiome.^[^ ^23^, ^24^, ^25^ ^]^

Moreover, the gut microbiota is implicated in arsenic‐induced toxicity. Several studies have shown that the gut microbiota can mitigate arsenic toxicity.^[^ ^26^, ^27^, ^28^ ^]^ A normal gut microbiome can alleviate metabolic disorders in mice under arsenic exposure, while more severe pathological changes and apoptosis are observed in multiple tissues, such as liver and kidney, when the gut microbiota is perturbed.^[^ ^27^, ^28^ ^]^ Recent reports have suggested that the gut microbiota contributes to fecal arsenic excretion and biotransformation,^[^ ^27^ ^]^ which provides a preliminary explanation for the beneficial effects of the gut microbiota. In addition to affecting arsenic metabolism, the gut microbiota may protect the host through other mechanisms.^[^ ^29^, ^30^, ^31^ ^]^ Oxidative stress and inflammation play essential roles in arsenic‐triggered adverse effects on the intestine, while the gut microbiota such as certain species that can produce short‐chain fatty acids (SCFAs) have immense antioxidative and anti‐inflammatory effects.^[^ ^32^, ^33^, ^34^ ^]^ Moreover, specific genera of the gut microbiota, such as *Akkermansia muciniphila*, can combat inflammatory bowel diseases by rebuilding mucus barriers, quelling inflammation, and restoring microbial balance.^[^ ^35^, ^36^, ^37^, ^38^ ^]^ Nevertheless, whether specific genera of the gut microbiota can withstand intestinal arsenic stress remains understudied.

In this study, we revealed that exposure to arsenic disrupted the intestinal barrier and changed the microenvironment in the intestines of mice. By using the fecal microbiota transplantation (FMT), our results verified that the administration of donor stool from healthy mice could alleviate the disruption of the intestinal barrier and alteration of the microenvironment in the intestine caused by arsenic. Importantly, after in vitro anaerobic culture, we discovered a new role of *Roseburia intestinalis* in mitigating the toxic effects of arsenic in the intestine. After metabolomics and transcriptomic analysis, we investigated the potential mechanisms involved in the beneficial effects of *Roseburia intestinalis* on arsenic‐induced intestinal toxicity. These findings provide novel insight into the underlying mechanisms of arsenic toxicity from the perspective of the gut microbiome and offer new clues for the prevention and treatment of arsenic‐related diseases.

## 2 Results

### 2.1 Exposure to Arsenic Disrupts the Mechanical Barrier of the Intestine in the Mice

Given that humans are usually exposed to arsenic via contaminated drinking water or food, the intestine is considered as the major target organ of arsenic.^[^ ^39^ ^]^ In previous work, we found that chronic exposure to arsenic disturbs the microenvironment in the intestine,^[^ ^40^ ^]^ but whether acute arsenic administration result in disruption of the intestinal structure and function has not been fully elucidated. Therefore, in this study, we generated an arsenic‐exposed animal model in C57BL/6J mice by the administration of arsenic for 2 weeks via drinking water to simulate the acute arsenic poisoning in the clinic.

**Figure 1:**
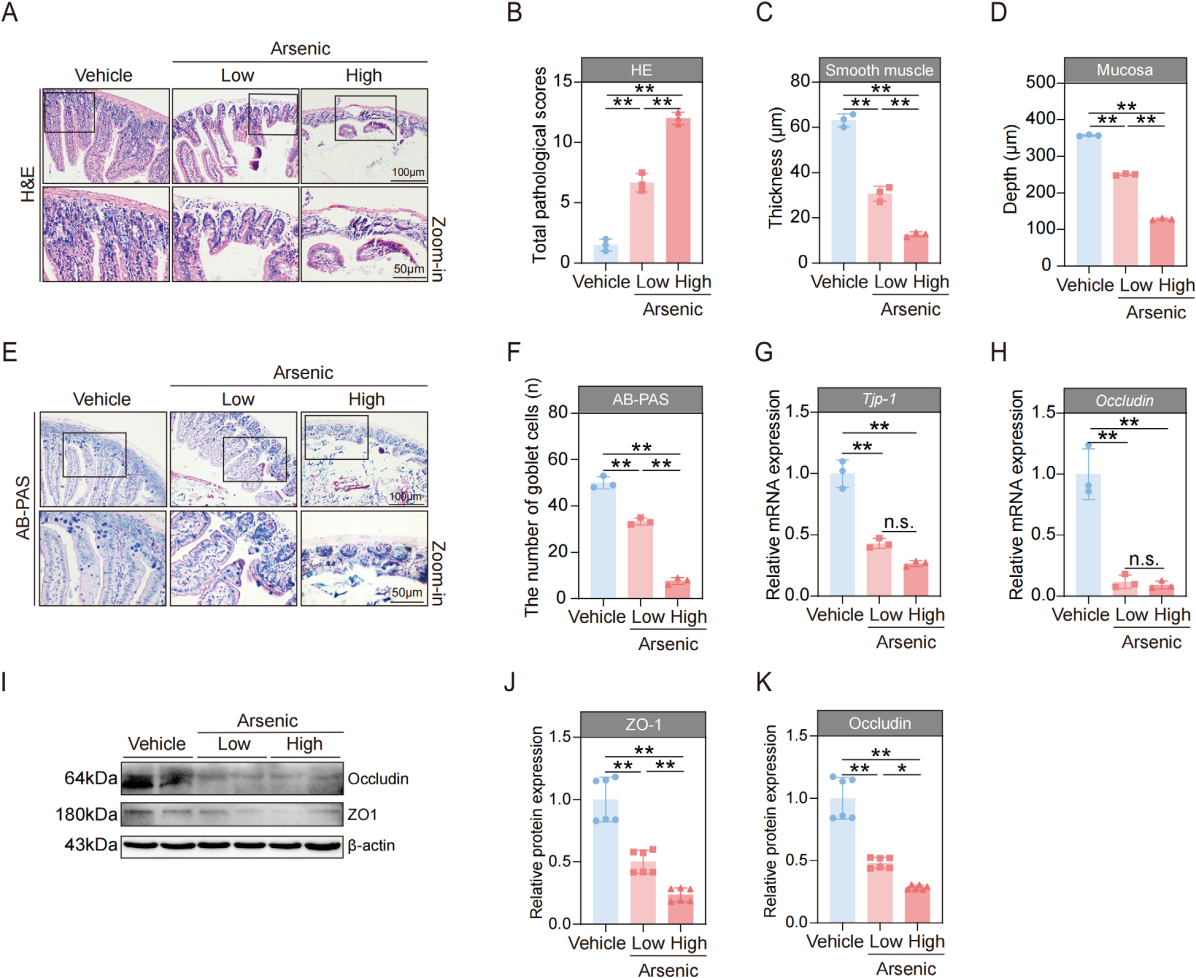
Arsenic disrupts the mechanical barrier of intestine in the mice. A) Representative hematoxylin and eosin (H&E) stained images of intestine tissues. Scale bars, 100 µm (top) and 50 µm (bottom). B–D) Total pathological score, smooth muscle thickness, and mucosa depth of the intestine tissues (*n* = 3). E) Representative Alcian Blue‐Periodic Acid‐Schiff (AB‐PAS) stained images of intestine tissues. Scale bars, 100 µm (top) and 50 µm (bottom). F) The number of goblet cells of the intestine tissues (*n* = 3). G,H) The mRNA expressions of *Tjp‐1* and *occludin* in intestines of mice (*n* = 3). I–K) Representative western blot images and statistical analyses of ZO‐1 and Occludin in intestines of mice (*n* = 2). Data are expressed as mean ± SD (B–D,F–H,J–K). ^*^ *p* < 0.05, ^**^ *p* < 0.01.

The intestinal barrier is composed of a mechanical barrier, chemical barrier, immune barrier, and biological barrier.^[^ ^41^ ^]^ First, we used hematoxylin and eosin (H&E) staining to assess the morphological changes in the intestine after treatment with arsenic. The results revealed that exposure to arsenic caused severe pathological changes in the intestine, the villi were obviously ruptured, and the lengths of the villi were decreased significantly. Moreover, these injuries in the intestine seemed to occur in a dose‐dependent manner, indicating that the high‐dose arsenic‐treated group exhibited much more serious histopathological alterations than the low‐dose group did; almost all the villi were ruptured after a high dose of arsenic was administrated (**Figure** **1**A). The pathological scores, smooth muscle thickness, and the mucosa length all markedly changed remarkably in a dose‐dependent manner (Figure 1B–D). Alcian Blue‐Periodic Acid‐Schiff (AB‐PAS) staining was subsequently used to evaluate the effects of arsenic on the number of goblet cells, which are a major type of intestinal mucosal epithelial cell that serves as the primary site for mucosal absorption.^[^ ^42^ ^]^ A significant decrease in the number of goblet cells was observed after arsenic exposure, particularly in the high‐dose group (Figure 1E,F). To further determine whether the integrity of the intestinal barrier is compromised by arsenic exposure, the expression of the tight junction proteins ZO‐1 and Occludin was measured at both the mRNA and protein levels. The results revealed that the mRNA expression of *Tjp‐1* and *oOccludin* dramatically decreased after arsenic exposure (Figure 1G,H). Moreover, similar trends were found for their protein expression levels (Figure 1I–K). Taken together, these findings suggest that the administration of arsenic disrupted the mechanical barrier of the intestine in the mice.

### 2.2 Exposure to Arsenic Affects the Immunological Barrier of the Intestine in the Mice

**Figure 2:**
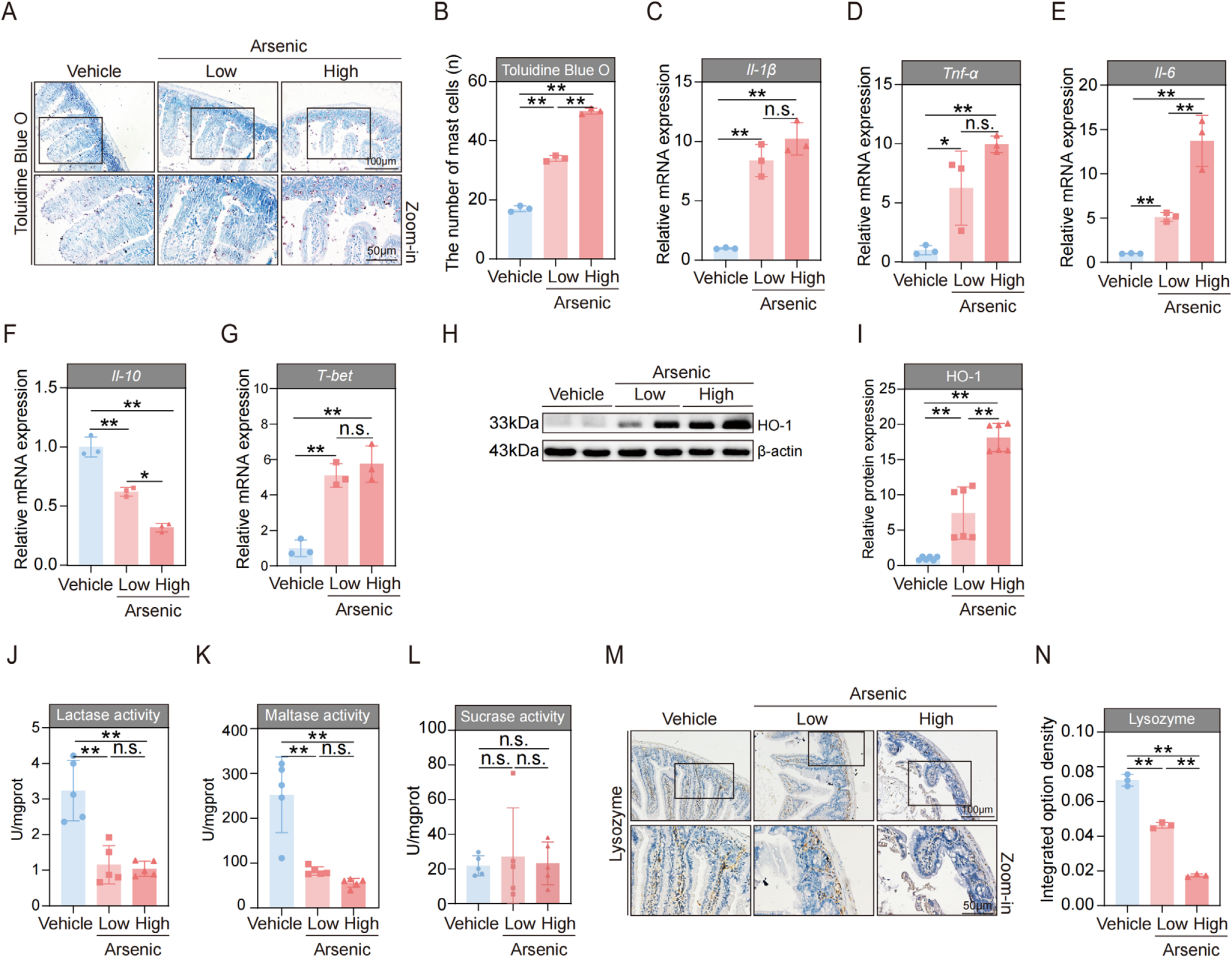
Arsenic affects the immunological barrier and chemical barrier of intestine in the mice. A) Representative Toluidine Blue O (TBO) stained images of intestine tissues. Scale bars, 100 µm (top) and 50 µm (bottom). B) The number of mast cells in the intestine tissues (*n* = 3). C–G) The mRNA expressions of *Il‐1β*, *Tnf‐α*, *Il‐6*, *Il‐10*, and *T‐bet* in intestines of mice (*n* = 3). H,I) Representative western blot images and statistical analyses of HO‐1 in intestines of mice (*n* = 2). J–L) The activities of intestinal lactase, maltase, and sucrase of mice (*n* = 5). M) Representative images of intestine tissues by immunohistochemistry staining of Paneth‐secreted protein lysozyme. Scale bars, 100 µm (top) and 50 µm (bottom). N) The integrated option density of lysozyme in intestines of mice (*n* = 3). Data are expressed as mean ± S.D. (B–G,I–L,N). ^*^ *p* < 0.05, ^**^ *p* < 0.01, n.s., no significance.

The immune barrier is another major gatekeeper of the intestine. It consists of intestinal epithelial cells, a large number of immune cells, and immune molecules dispersed in the intestinal mucosal epithelium and lamina propria, as well as gut‐associated lymphoid tissue. In this study, we used Toluidine Blue O (TBO) staining to evaluate the effect of arsenic on the number of mast cells, which are constitutively found in the intestine and can modulate both innate and adaptive mucosal immunity. Our results revealed that the administration of arsenic significantly elevated mast cell counts, particularly in the high‐dose group (**Figure** **2**A,B). In addition, arsenic profoundly increased the expression of *Il‐1β*, *Tnf‐α*, and *Il‐6* in the intestine, indicating that an imbalance in immune homeostasis occurred after exposure (Figure 2C–E). The increase in these inflammatory indicators caused by arsenic also occurred in a relative dose‐dependent manner. As depicted in the Figure 2F, we observed the opposite trend in the mRNA expression of *Il‐10*, which is an anti‐inflammatory cytokine that maintains the balance of the immune response. Since T‐bet plays a crucial role in regulating mucosal immune responses, we next assessed the mRNA expression of *T‐bet* after designed treatment with arsenic. The data revealed that exposure to arsenic dramatically elevated the level of *T‐bet* in the intestine (Figure 2G). HO‐1 not only exerts anti‐inflammatory effects but also plays a beneficial role in protecting the intestinal mucosal barrier. Similar to the expression of *T‐bet*, exposure to arsenic significantly elevated the protein expression of HO‐1 (Figure 2H,I). Taken together, these above results suggest that arsenic treatment of arsenic disturbs the balance of immune homeostasis in the intestine as well as the immunological barrier of the intestine in mice.

### 2.3 Exposure to Arsenic Disturbs the Chemical Barrier of the Intestine in Mice

The digestive enzymes are predominantly present in the intestine and are involved in forming the intestinal chemical barrier.^[^ ^43^ ^]^ Therefore, we first assessed the activities of lactase, maltase and sucrase after the treatment of the mice with or without arsenic. As presented in Figure 2J,K, compared with those of the vehicle control, the lactase and maltase activities were markedly inhibited by arsenic, in both the low‐dose and high‐dose groups. However, no significant differences in sucrase activities were detected between the arsenic‐treated animals and vehicle control animals (Figure 2L). Paneth cells are a major type of intestinal epithelial cell located in the crypts of the intestine and they secrete antimicrobial proteins, such as lysozyme, into the intestinal lumen, which compromises the intestinal chemical barrier.^[^ ^44^ ^]^ Thus, we determined the expression level of lysozyme after the administration of arsenic. Our results revealed that lysozyme‐secreting Paneth cells also abnormally declined in response to arsenic exposure (Figure 2M,N), indicating the loss of Paneth cells and their antimicrobial granules. These results imply that exposure to arsenic disturbs the chemical barrier of the intestine in mice.

### 2.4 Exposure to Arsenic Influences the Biological Barrier of the Intestine in Mice

**Figure 3:**
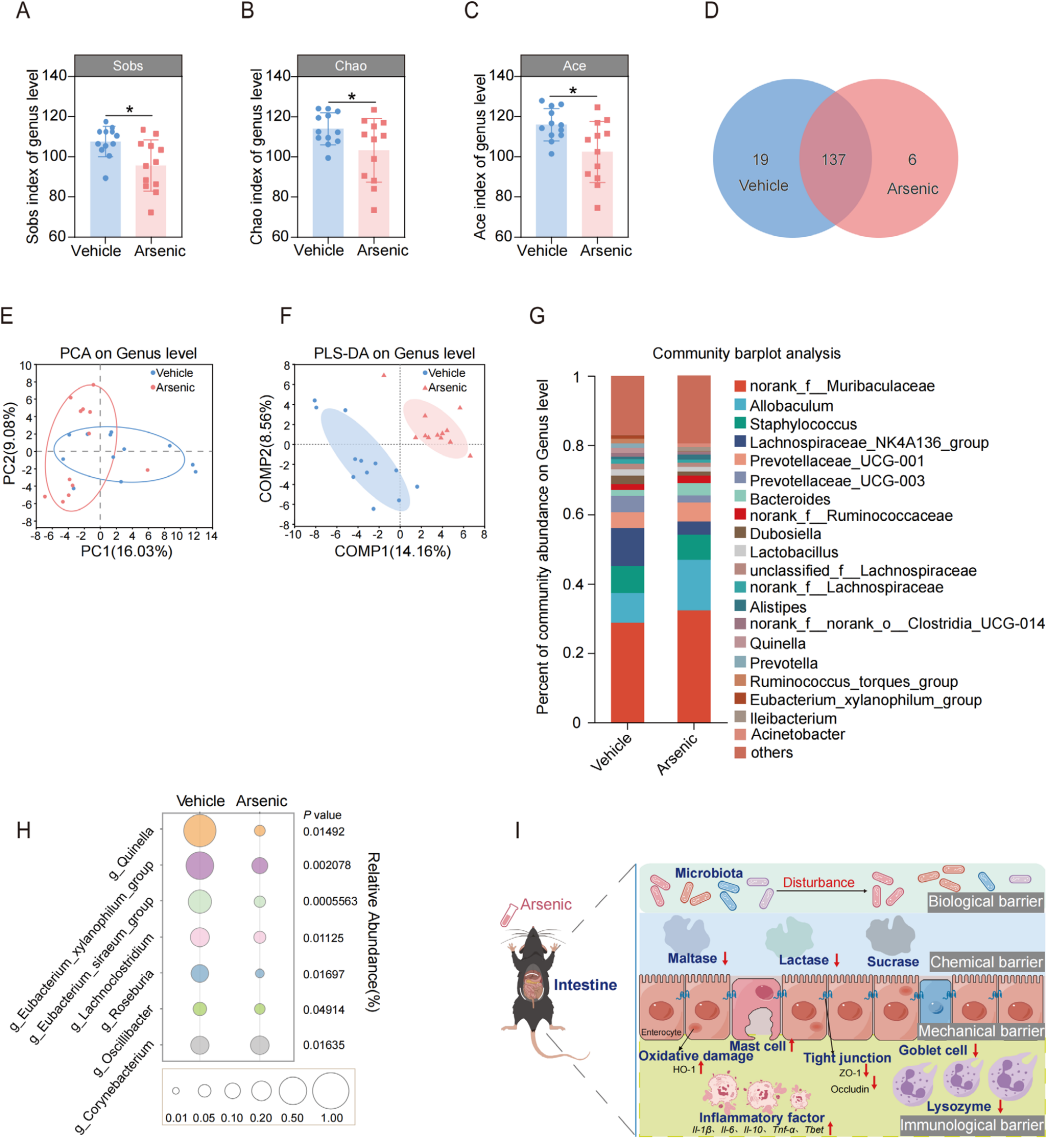
Arsenic influences the biological barrier of intestine in the mice. A–C) Alpha diversity of gut microbiota assessed by Sobs, Chao, and Ace indexes (*n* = 12). D) Venn diagram based on genus level. E,F) Principal component analysis (PCA) and partial least squares discriminant analysis (PLS‐DA) score plots for gut microbiota based on genus level. G) Mean relative abundance gut microbiota based on genus level. H) Bubble chart for comparison of the top seven altered microbial induced by arsenic. I) Schematic diagram of the arsenic‐induced adverse effects on the intestinal biological, chemical, mechanical, and immunological barriers. Data are expressed as mean ± S.D. (A–C). ^*^ *p* < 0.05.

The gut microbiota is essential for the maintenance of intestinal barrier integrity and function. Thus, it is considered the biological barrier in the intestine.^[^ ^43^ ^]^ In this study, we found that exposure to arsenic caused significant alterations in the richness and evenness of the gut microbiome of mice. After data quality filtration, there were 1118938 high‐quality sequences remained from a total of 1281218. As shown in **Figure** **3**A–C, the α‐diversity indices Sobs, Chao, and Ace were lower in the arsenic group than in the vehicle control group at the genus level, indicating that community richness and evenness were reduced after arsenic administration. No significant difference was found in the coverage index between groups, indicating that the sequencing depth was adequate to ensure data accuracy (Figure S1A, Supporting Information). To our surprise, we did not observe any alterations in the Shannon or Simpson indices at the genus level (Figure S1B,C, Supporting Information).

The numbers of shared and unique genera between the control and arsenic‐treated groups were visualized by a Venn diagram (Figure 3D). Specifically, 137 core genera were shared, 19 unique genera were present in the control group, and 6 unique genera were present in the arsenic group. To further evaluate the community composition of the gut microbiota, both principal component analysis (PCA) and partial least squares discrimination analysis (PLS‐DA) were performed at the genus level. Our results demonstrated that the administration of arsenic caused a significant shift in the gut microbiota composition (Figure 3E,F). The percentages of community abundance at the genus level were shown in Figure 3G. Specifically, compared with those in the vehicle group, the abundances of the seven most abundant gebera, *g*_*Quinella*, *g*_*Eubacterium_xylanophilum_group*, *g*_*Eubacterium_ siraeum_group*, *g*_*Lachnoclostridium*, *g*_*Roseburia*, *g*_Oscillibacter, and *g*_ *Corynebacterium* were markedly decreased at the genus level (Figure 3H). These data collectively suggest that exposure to arsenic via drinking water disturbs the biological barrier in the intestines of mice. The adverse effects of arsenic on the biological, chemical, mechanical and immunological barriers were summarized in Figure 3I.

### 2.5 Fecal Microbiota Transplantation Ameliorated the Harmful Effects of Arsenic in the Intestines of Mice

**Figure 4:**
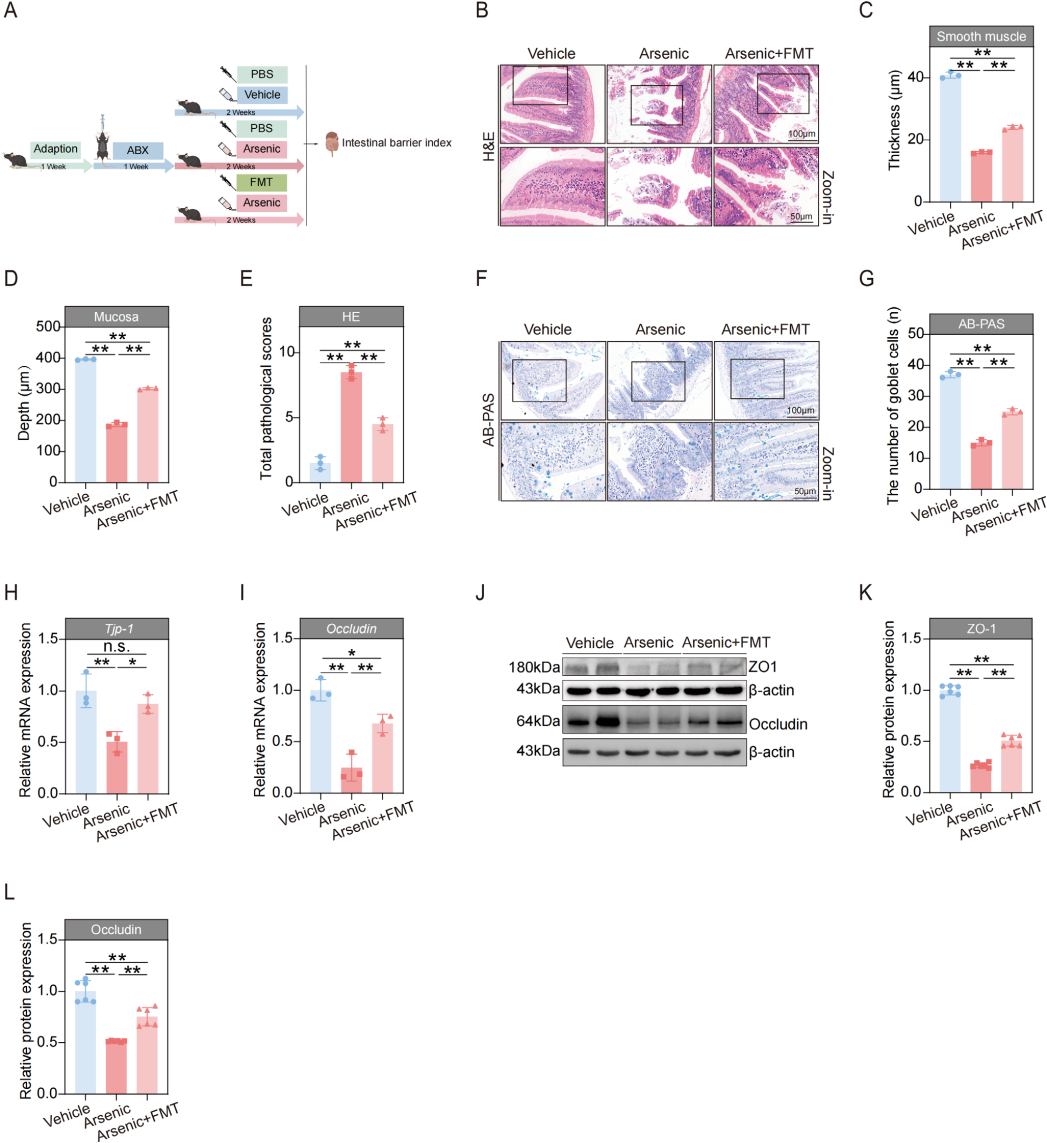
Fecal microbiota transplantation (FMT) ameliorated the adverse effects of arsenic on the intestinal mechanical barrier of mice. A) Schematic diagram of the FMT experimental design. B) Representative hematoxylin and eosin (H&E) stained images of intestine tissues. Scale bars, 100 µm (top) and 50 µm (bottom). C–E) Smooth muscle thickness, mucosa depth, and the total pathological score of the intestine tissues (*n* = 3). F) Representative Alcian Blue‐Periodic Acid‐Schiff (AB‐PAS) stained images of intestine tissues. Scale bars, 100 µm (top) and 50 µm (bottom). G) The number of goblet cells of the intestine tissues (*n* = 3). H,I) The mRNA expressions of *Tjp‐1* and occludin in intestine of mice (*n* = 3). J–L) Representative western blot images and statistical analyses of ZO‐1 and Occludin in intestines of mice (*n* = 2). Data are expressed as mean ± SD (C–E, J–I, K,L). ^*^ *p* < 0.05, ^**^ *p* < 0.01.

To clarify the role of the gut microbiota in the regulation of arsenic‐induced intestinal barrier impairments, the fecal microbiota transplantation (FMT) was conducted because this method is an underutilized, widely available, and inexpensive tool used to connect changes in gut microbes with many pathologies.^[^ ^45^ ^]^ The detailed study design is shown in **Figure** **4**A. After the fecal microbiota derived from healthy mice was transplanted, the intestinal tissue damage caused by arsenic was markedly ameliorated, manifested by increased smooth muscle thickness (Figure 4B,C), increased mucosa depth (Figure 4D), decreased total pathological scores (Figure 4E), and an increased number of goblet cells (Figure 4F,G), as shown by H&E and AB‐PAS staining. Moreover, the decreases in the mRNA and protein expression levels of ZO‐1 and Occludin were alleviated by the administration of FMT with fecal obtained from healthy mice (Figure 4H–L). These results suggest that FMT is an effective way to treat arsenic‐induced disruption of the mechanical barrier of the intestines in mice.

**Figure 5:**
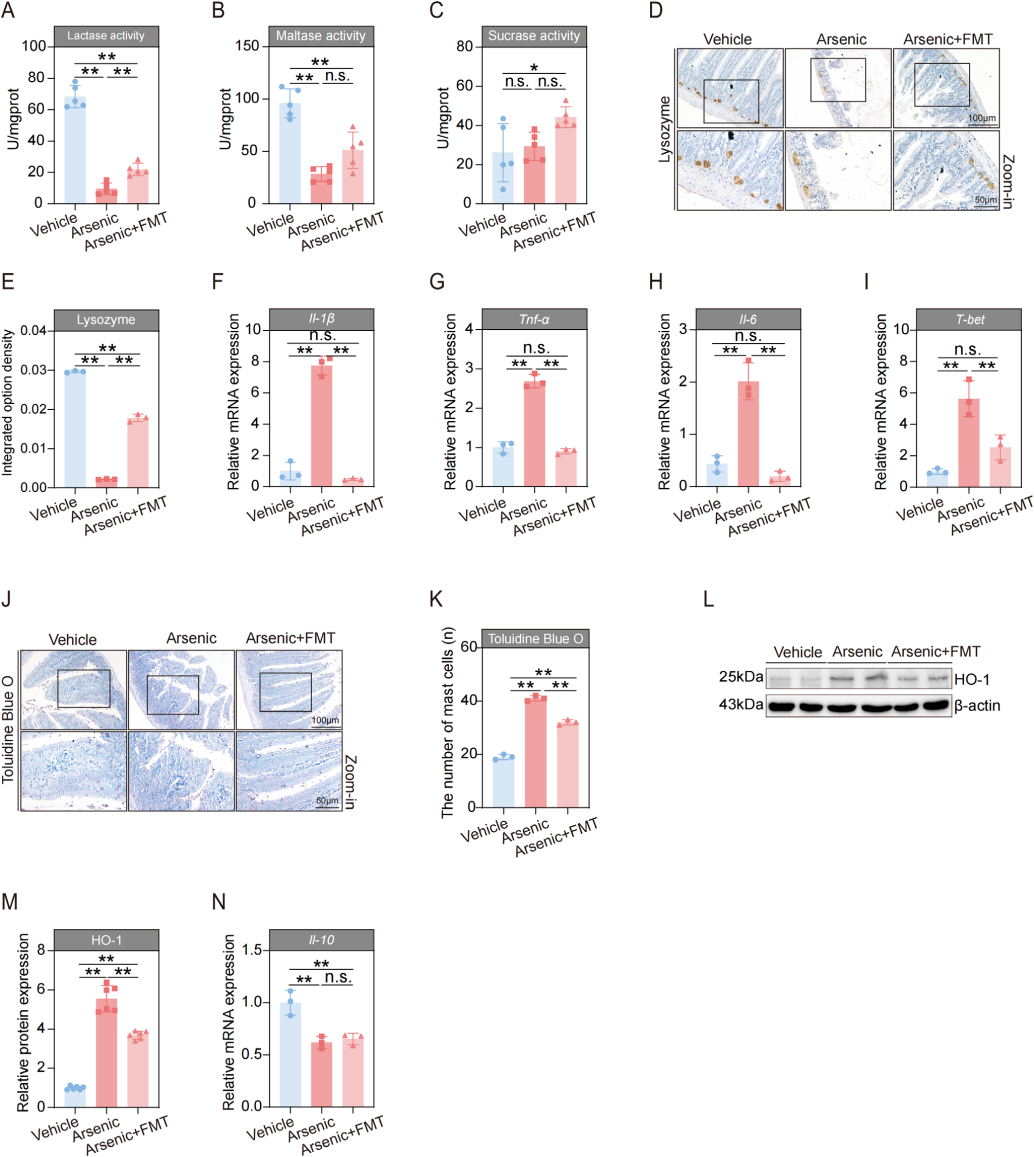
Fecal microbiota transplantation (FMT) ameliorated the harmful effects of arsenic on the intestinal immunological barrier and chemical barrier of mice. A–C) The activities of intestinal lactase, maltase, and sucrase of mice (*n* = 5). D) Representative images of intestine tissues by immunohistochemistry staining of Paneth‐secreted protein lysozyme. Scale bars, 100 µm (top) and 50 µm (bottom). E) The integrated option density of lysozyme in intestines of mice (*n* = 3). F–I) The mRNA expressions of *Il‐1β*, *Tnf‐α*, *Il‐6*, and *T‐bet* in intestines of mice (*n* = 3). J) Representative Toluidine Blue O (TBO) stained images of intestine tissues. Scale bars, 100 µm (top) and 50 µm (bottom). K) The number of mast cells in the intestine tissues (*n* = 3). L,M) Representative western blot images and statistical analyses of HO‐1 in intestines of mice (*n* = 2). N) The mRNA expressions of *Il‐10* in intestines of mice (*n* = 3). Data are expressed as mean ± S.D. (A–C,E–I,K,M,N). ^*^ *p* < 0.05, ^**^ *p* < 0.01, n.s., no significance.

We then assessed the indicators of the chemical barrier of the intestine. As expected, the activity of lactase was obviously greater in the arsenic + FMT group than in the arsenic‐treated group (**Figure** **5**A). Compared with the arsenic‐administered group, the maltase activity in the arsenic + FMT group slightly increased, although the difference did not reach formal statistical significance (Figure 5B). However, no significant difference was found in the level of sucrase activity among the three groups (Figure 5C). Similar trends were observed for the expression of lysozyme, showing that FMT treatment significantly alleviated the decrease in the number of lysozyme‐secreting Paneth cells (Figure 5D,E). Taken together, these results together suggest that FMT with healthy fecal microbiota alleviates the degree of injury to the chemical barrier of the intestine.

Next, we further evaluated the indicators of the immunological barrier of the intestine. The increases in the mRNA expression levels of *Il‐1β*, *Tnf‐α*, *Il‐6*, and *T‐bet* caused by arsenic were all sharply reduced after FMT with healthy fecal microbiota (Figure 5F–I). Similar trends were observed in the number of mast cells stained with TBO (Figure 5J,K) and the protein expression of HO‐1 (Figure 5L,M). However, to our surprise, the arsenic‐induced decrease in the expression of *Il‐10* by arsenic was not be attenuated by FMT (Figure 5N). These results indicate that FMT with healthy gut microbiota partially restores the immune barrier in the intestines of arsenic‐exposed mice.

### 2.6 *Roseburia Intestinalis* Effectively Attenuated the Adverse Effects of Arsenic in the Intestines of Mice

To further establish potentially bioactive bacterial strains in the FMT mixture, we carefully analyzed the results of 16S rRNA gene sequencing at the genus level. As one of the seven bacteria whose abundance significantly decreased in response to arsenic, the abundance of *Roseburia* was obviously reduced by arsenic administration. The genus *Roseburia* is of particular interest, and includes five species: *Roseburia intestinalis*, *Roseburia hominis*, *Roseburia inulinivorans*, *Roseburia faecis*, and *Roseburia cecicola*.^[^ ^46^ ^]^ Among these five species, *Roseburia intestinalis* is a dominant symbiont of the human gut microbiota that produces SCFAs, which have many health benefits (La et al.^[^ ^47^ ^]^). More importantly, there is increasing evidence that *Roseburia intestinalis* has positive effects on several diseases, including inflammatory bowel disease, and is considered a potential probiotic.^[^ ^48^ ^]^ Therefore, *Roseburia intestinalis* was chosen and used for further experiments. Other top‐altered bacteria were not selected because *g_Quinella* is characterized primarily as a rumen‐specific bacterium with no available cultured strains or standardized murine models^[^ ^49^ ^]^; *Eubacterium_xylanophilum's* has been linked to neurological disorders and *Eubacterium_siraeum's* undefined role in SCFA production/immune modulation lacks direct evidence for arsenic‐induced gut injury^[^ ^50^, ^51^ ^]^; *Corynebacterium* includes opportunistic pathogens unsuitable for therapy, and *Lachnoclostridium's* species‐level functional duality (probiotic versus pathogenic) creates ambiguity.^[^ ^52^, ^53^, ^54^ ^]^

**Figure 6:**
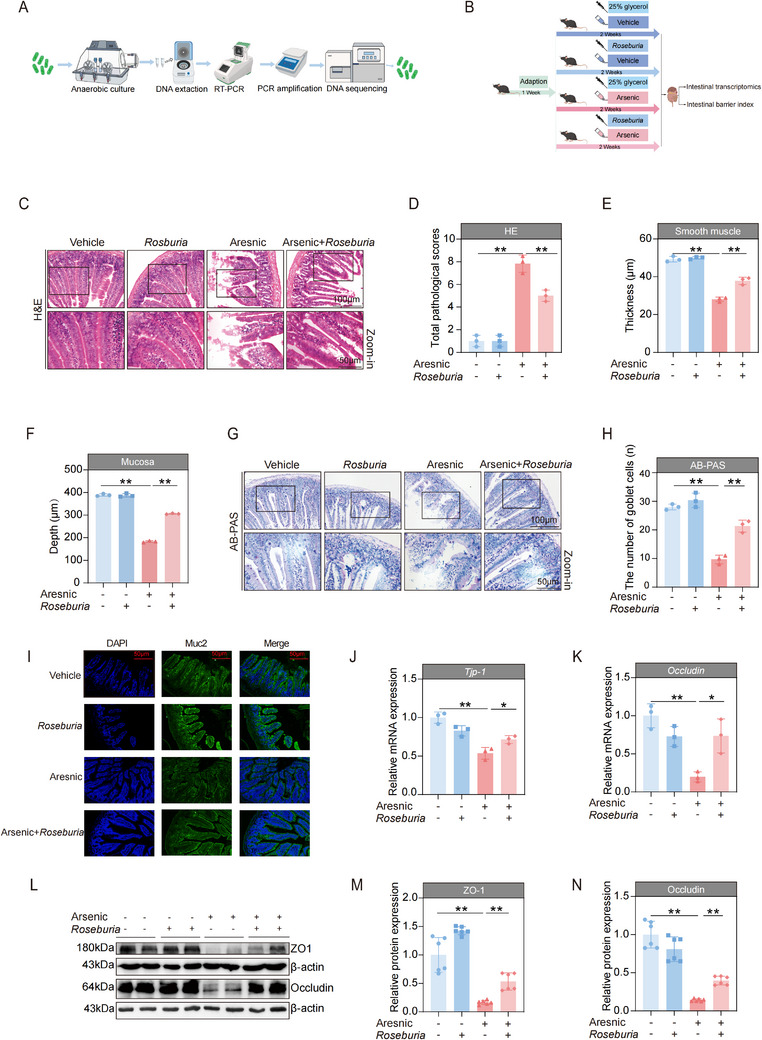
*Roseburia intestinalis* effectively attenuated the adverse effects of arsenic on the intestinal mechanical barrier of mice. A) Schematic diagram of *Roseburia intestinalis* culture and verification. B) Schematic diagram of the experimental design about *Roseburia intestinalis* administration. C) Representative hematoxylin and eosin (H&E) stained images of intestine tissues. Scale bars, 100 µm (top) and 50 µm (bottom). D–F) The total pathological score, smooth muscle thickness, and mucosa depth of the intestine tissues (*n* = 3). G) Representative Alcian Blue‐Periodic Acid‐Schiff (AB‐PAS) stained images of intestine tissues. Scale bars, 100 µm (top) and 50 µm (bottom). H) The number of goblet cells of the intestine tissues (*n* = 3). I) Representative images Muc2 expression in the small intestine of mice detected by immunofluorescence. Scale bar: 50 µm (*n* = 3). J,K) The mRNA expressions of *Tjp‐1* and occludin in intestines of mice (*n* = 3). L–N) Representative western blot images and statistical analyses of ZO‐1 and Occludin in intestines of mice (*n* = 2). Data are expressed as mean ± SD (D–F,H,J,K,M,N). ^*^ *p* < 0.05, ^**^ *p* < 0.01.

**Figure 7:**
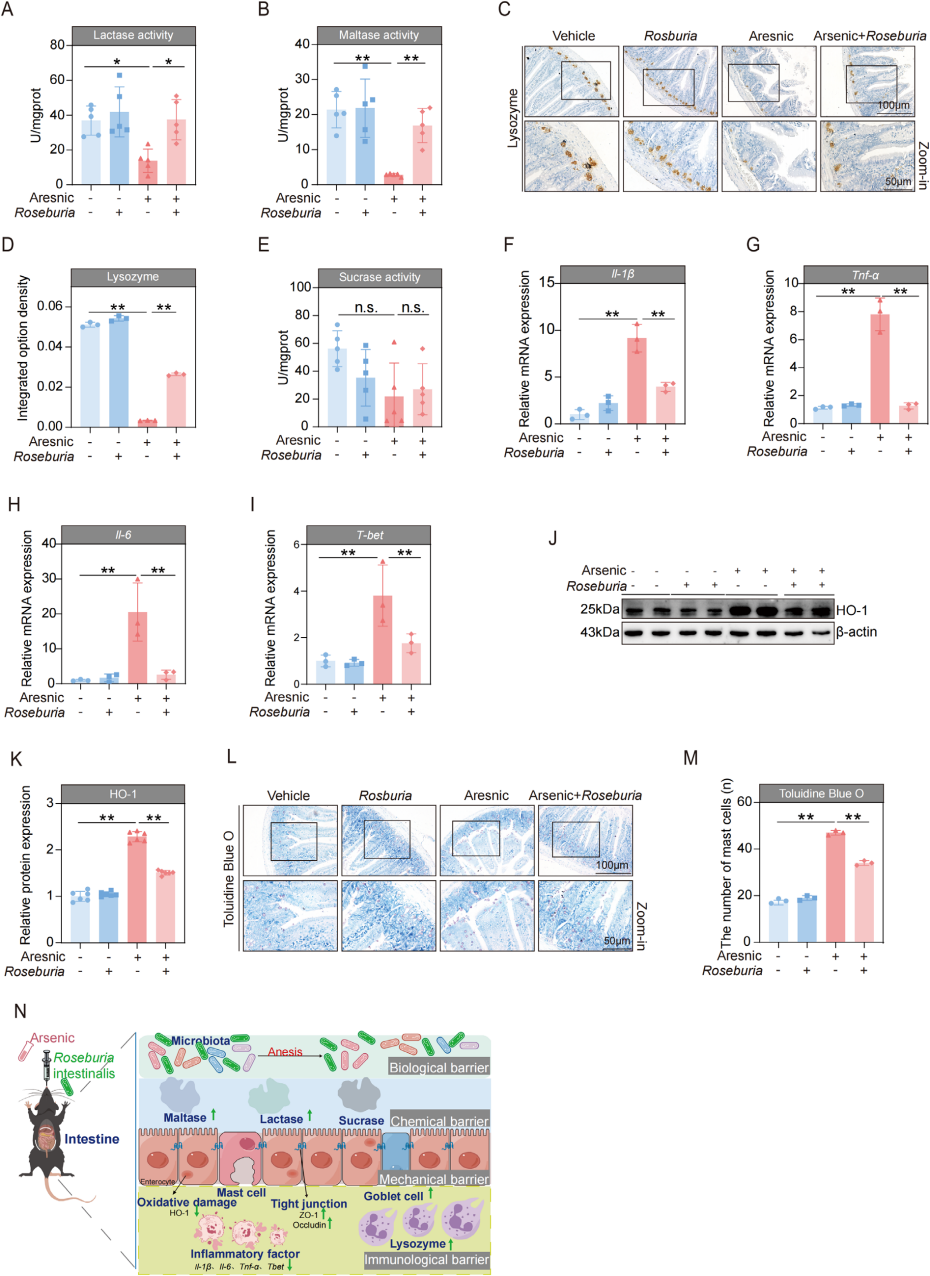
*Roseburia intestinalis* effectively attenuated the adverse effects of arsenic on the intestinal immunological barrier and chemical barrier of mice. A,B) The activities of intestinal lactase and maltase of mice (*n* = 5). C) Representative images of intestine tissues by immunohistochemistry staining of Paneth‐secreted protein lysozyme. Scale bars, 100 µm (top) and 50 µm (bottom). D) The integrated option density of lysozyme in intestines of mice (*n* = 3). E) The activity of intestinal sucrase of mice (*n* = 5). F–I) The mRNA expressions of *Il‐1β*, *Tnf‐α*, *Il‐6*, and *T‐bet* in intestines of mice (*n* = 3). J,K) Representative western blot images and statistical analyses of HO‐1 in intestines of mice (*n* = 2). L) Representative Toluidine Blue O (TBO) stained images of intestine tissues. Scale bars, 100 µm (top) and 50 µm (bottom). M) The number of mast cells in the intestine tissues (*n* = 3). N) Schematic diagram of the protective effect of gut‐derived *Roseburia intestinalis* on arsenic‐induced intestinal injuries. Data are expressed as mean ± S.D. (A,B,D–I,K,M). ^*^ *p* < 0.05, ^**^ *p* < 0.01, n.s., no significance.

*Roseburia intestinalis* was cultured under anaerobic culture conditions and verified by using DNA sequencing (**Figure** **6**A). According to the flow cytometry results, the viability of the in vitro cultivated *Roseburia intestinalis* was greater than 93% (Figure S2, Supporting Information). The study design for the administration of *Roseburia intestinalis* was shown in Figure 6B. Compared with that in the control group, the abundance of *Roseburia intestinalis* was significantly increased after *Roseburia intestinalis* gavage. Similar results were observed in the arsenic‐exposed mice, as higher *Roseburia intestinalis* abundance was found in the mice that received *Roseburia intestinalis* (Figure S3, Supporting Information). H&E staining revealed that supplementation with *Roseburia intestinalis* effectively ameliorated the increased total pathological scores (Figure 6C,D) and decreased the smooth muscle thickness (Figure 6E) and mucosal depth (Figure 6F) induced by arsenic. In the AB‐PAS staining revealed that decrease in the number of goblet cells induced by arsenic was also attenuated by treatment with *Roseburia intestinalis* (Figure 6G,H). Mucin 2 (Muc2) is a key component of mucous that is synthesized and secreted by epithelial goblet cells. In parallel with the goblet cell counts observed by a AB‐PAS staining, higher Muc2 expression levels were detected in the arsenic+ *Roseburia intestinalis* group than in the arsenic group (Figure 6I). What's more, the decreases in the mRNA and protein expression levels of ZO‐1 and Occludin were both attenuated by *Roseburia intestinalis* (Figure 6J–N). Similarly, we observed that treatment with *Roseburia intestinalis* profoundly attenuated the decreased activities of lactase and maltase induced by arsenic (**Figure** **7**A,B). Moreover, supplementation with *Roseburia intestinalis* markedly reversed the decline in the expression of lysozyme caused by arsenic (Figure 7C,D). Nonetheless, no significant effect on the activity of sucrase was detected (Figure 7E).

For immunological barrier factors, the results revealed that the elevated mRNA expressions of *Il‐1β*, *Tnf‐α*, *Il‐6*, and *T‐bet* caused by arsenic were all significantly decreased after treatment with *Roseburia intestinalis* (Figure 7F–I). Furthermore, increase in HO‐1 protein expression induced by arsenic was diminished after *Roseburia intestinalis* administration (Figure 7J,K). A similar trend was also found on the number of mast cells (Figure 7L,M). Importantly, the administration of *Roseburia intestinalis* did not induce any apparent side effects. Taken together, these findings indicate that gut‐derived *Roseburia intestinalis* plays a protective role against arsenic‐induced intestinal injury (summarized in Figure 7N).

### 2.7 Screen of Differentially Expressed Metabolites Related to *Roseburia Intestinalis* After Arsenic Exposure in Mice

**Figure 8:**
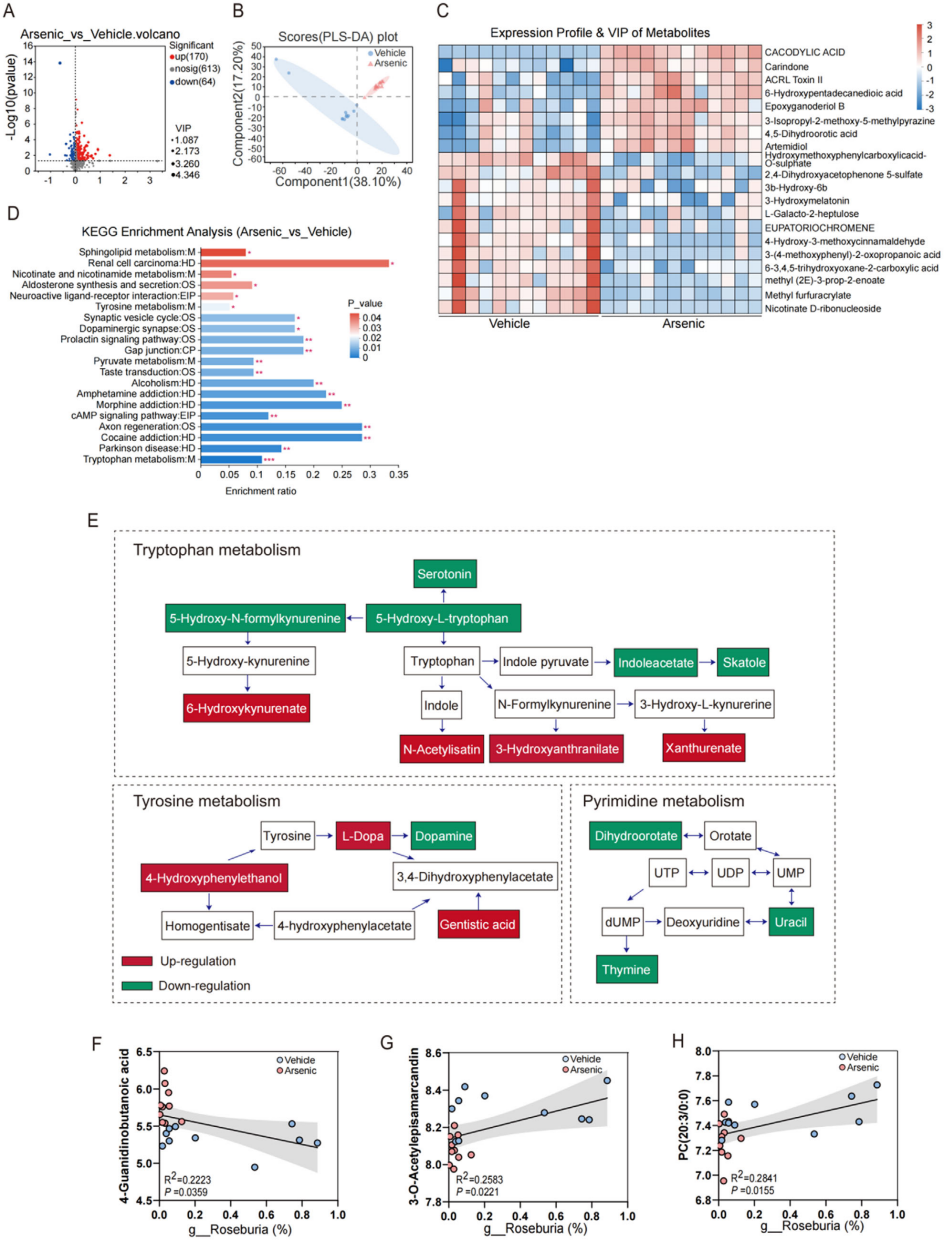
*Roseburia intestinalis* caused changes in metabolites of serum perturbed by arsenic. A) Volcano plot of metabolomics data in serum of mice. B) Partial least squares discriminant analysis (PLS‐DA) score plot on the metabolic profiles in serum of mice. C) Heatmap of the top 20 differently expressed metabolites. D) Kyoto Encyclopedia of Genes and Genomes (KEGG) pathway enrichment analysis of differently expressed metabolites. E) The important metabolic pathways influenced by arsenic. F–H) Spearman's correlations between *Roseburia intestinalis* abundance and the major differently expressed metabolites. *n* = 10.

Previous studies have reported that gut‐derived bacteria usually generate specific metabolites and these mediate their influences on host health and pathophysiological functions.^[^ ^55^ ^]^ To further establish the potential metabolites associated with *Roseburia intestinalis* and its beneficial effects, we then conducted nontargeted metabolomics analysis after arsenic exposure. As presented in the volcano plot in **Figure** **8**A, a total of 234 distinct metabolites were identified between the arsenic group and vehicle group. Specifically, 170 metabolites were significantly increased, whereas 64 metabolites were reduced by arsenic. PLS‐DA scores differed significantly between the arsenic group and the control group, indicating plasma metabolite levels were significantly altered after arsenic exposure (Figure 8B). The top 20 differentially expressed metabolites were shown in a cluster heatmap. Eight metabolites were significantly increased in the arsenic group, including cacodylic acid, Carindone, acrl toxin II, and 6‐hydroxypentadecanedioic acid. Twelve metabolites were significantly decreased, including 3‐hydroxymelatonin, l‐galacto‐2‐heptulose, methyl furfuracrylate, and nicotinate D‐ribonucleoside etc. (Figure 8C). Furthermore, Kyoto Encyclopedia of Genes and Genomes (KEGG) enrichment analysis revealed that 20 metabolic pathways, including those related to sphingolipid metabolism, renal cell carcinoma, nicotinate metabolism, and nicotinamide metabolism, were apparently altered by arsenic exposure (Figure 8D). Notably, many of the differentially expressed metabolites are involved in tryptophan metabolism. As shown in Figure 8E, exposure to arsenic increased the contents of 6‐hydroxykynurenate, *N*‐acetylisatin, 3‐hydroxyanthranilate, and xanthurenate, but decreased the contents of 5‐hydroxy‐l‐tryptophan, serotonin, 5‐hydroxy‐*N*‐formylkynurenine, indoleacetate, and skatole.

Next, we analyzed the correlation between the abundance of *Roseburia intestinalis* and the identified differentially expressed metabolites. The major correlation plots depicted in Figure 8F–H and Figure S4 (Supporting Information), showing that the metabolites 3‐*O*‐acetylepisamarcandin, PC(20:3/0:0), and formiminoglutaminc acid were positively correlated with the abundance of *Roseburia intestinalis*, whereas other metabolites, such as 4‐guanidinobutanoic acid and L‐proline were negatively correlated with its abundance. Nevertheless, no significant correlations were detected between the abundance of Roseburia intestinalis and *N*‐decanoylglycine, enkephalin L, 12(R)‐HETE, or vinylacetylglycine. These results suggest the protective effects of *Roseburia intestinalis* may occur through the modulation of the metabolism of some of these metabolites upon arsenic exposure.

### 2.8 Screen of Genes with Differential Expression Related to *Roseburia Intestinalis* After Arsenic Exposure in Mice

**Figure 9:**
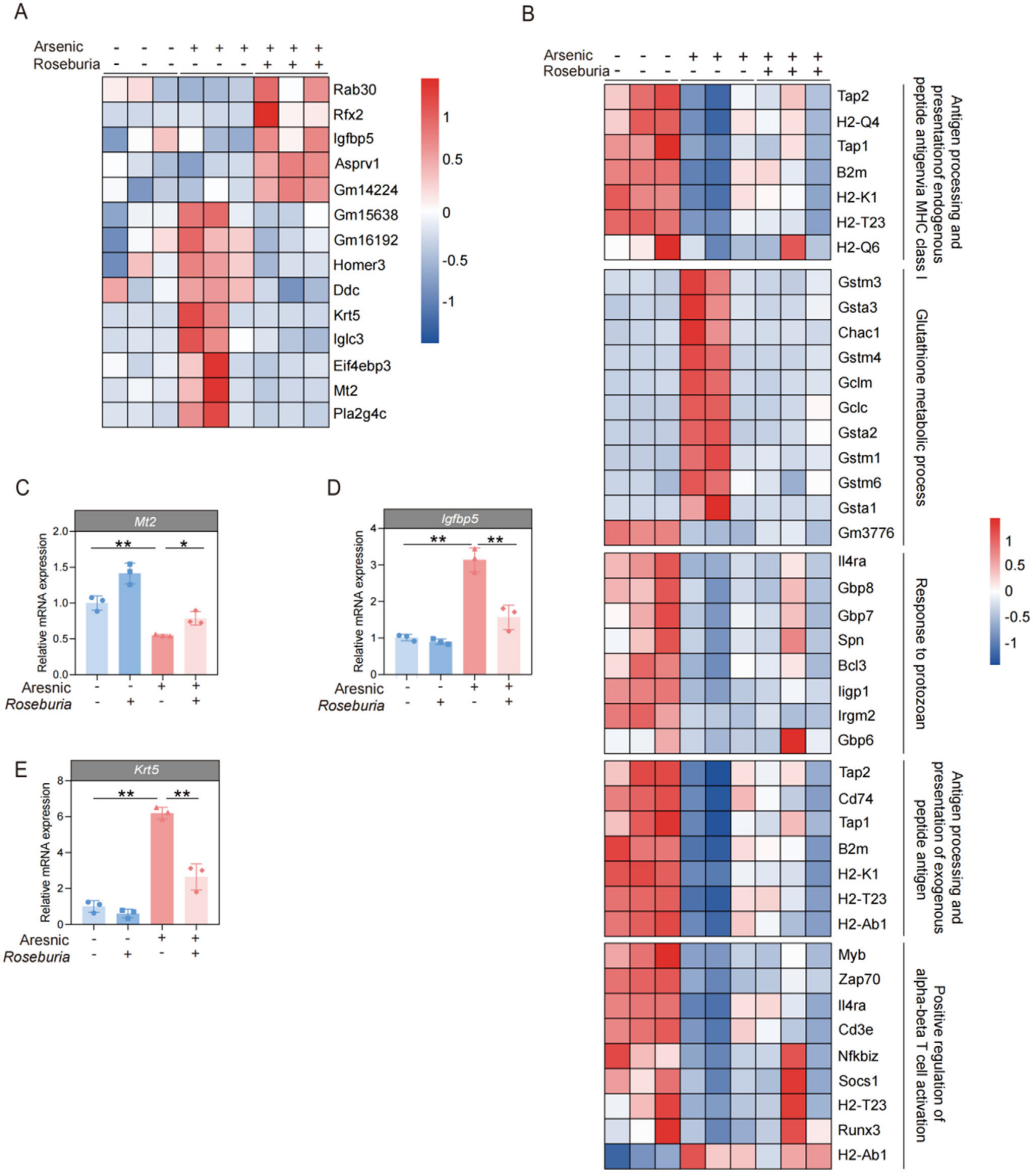
*Roseburia intestinalis* caused changes in intestinal gene expression perturbed by arsenic. A) Heatmap of the differently expressed genes in the intestine tissues of mice. B) Kyoto Encyclopedia of Genes and Genomes (KEGG) pathway enrichment analysis of differently expressed genes. C–E) The mRNA expression levels of *Igfbp5*, *Krt5*, and *Mt2* in intestines of mice, detected by RT‐qPCR (*n* = 3). Data are expressed as mean ± S.D.^*^ *p *< 0.05, ^**^ *p* < 0.01.

To further determine the potential genes involved in the protective effects of *Roseburia intestinalis* against arsenic‐induced intestinal injury, transcriptomic analysis of intestinal tissue was conducted. A total of 541 genes whose expression significantly changed by at least twofold were identified. Among these genes, 232 genes were upregulated, whereas 309 genes were downregulated after arsenic exposure in intestinal tissues. After cotreatment with *Roseburia intestinalis* and arsenic, we identified 14 significantly changed genes, including *Rab30*, *Rfx2*, *Lgfbp5*, and *Asprv1* (**Figure** **9**A). The results of the KEGG pathway analysis results revealed that the protective effects of *Roseburia intestinalis* against arsenic‐induced intestinal injury mainly included antigen processing and presentation of endogenous peptide antigens via MHC class I, antigen processing and presentation of exogenous peptide antigen, glutathione metabolic processes, positive regulation of alpha‐beta T‐cell activation, and response to protozoans (Figure 9B).

By combining literature reports with the results of hub gene analysis, three representative genes namely, *Igfbp5*, *Krt5*, and *Mt2* were selected for further verification using qPCR. As shown in Figure 9C, increased expression of *Igfbp5* and *Krt5*, and decreased expression of *Mt2* were detected after arsenic exposure. After *Roseburia intestinalis* administration, the increase in the mRNA expressions of *Igfbp5* and *Krt5* caused by arsenic were significantly decreased, whereas the decrease in the mRNA expressions of *Mt2* caused by arsenic was significantly increased. The protein encoded by the *Igfbp5* belongs to the IGFBP family and primarily regulates the bioavailability of insulin‐like growth factors. Recent studies have revealed that elevated *Igfbp5* expression is closely linked to the exacerbation of intestinal inflammation and disruption of gut barrier integrity.^[^ ^56^, ^57^ ^]^ The *Krt5* gene encodes Keratin 5, which is a critical structural protein of the epithelial cytoskeleton. KRT5 deficiency compromises cytoskeletal stability, however, existing research has primarily focused primarily on the colon, and whether there is functional heterogeneity across intestinal segments still needs further study.^[^ ^58^ ^]^ MT2 is rich in cysteine residues and can mitigate toxicity to intestinal epithelial cells by scavenging reactive oxygen species and counteracting inflammation.^[^ ^59^, ^60^ ^]^ These results imply that the beneficial effect of *Roseburia intestinalis* may be exerted through transcriptomic alternations, such as modulating the expression levels of *Igfbp5*, *Krt5*, and *Mt2*, thus mitigating inflammation and ultimately preserving gut barrier integrity.

### 2.9 Culture Supernatant of *Roseburia Intestinalis* Alleviated the Toxic Effects of Arsenic on the Caco‐2 and HT‐29 Intestinal Cell Lines

The Caco‐2 and HT‐29 intestinal cell lines were used to elucidate the potential mechanism by which *Roseburia intestinalis* exerts its protective effect. As shown in Figure S5 (Supporting Information), no obvious changes in cell viability were detected when the arsenic dose was 2.5  µmol, whereas when the arsenic dose reached 5  µmol, the viability of Caco‐2 and HT‐29 cells was significantly reduced. Therefore, the cells were treated with 5  µmol of arsenic in the following experiments. Moreover, the results of the dose‐response optimization assays indicated that a 1:1 dilution (v/v) of *Roseburia intestinalis culture supernatant* with fresh cell medium was the optimal dose of *Roseburia intestinalis* intervention in the in vitro experiments (Figure S6, Supporting Information).

**Figure 10:**
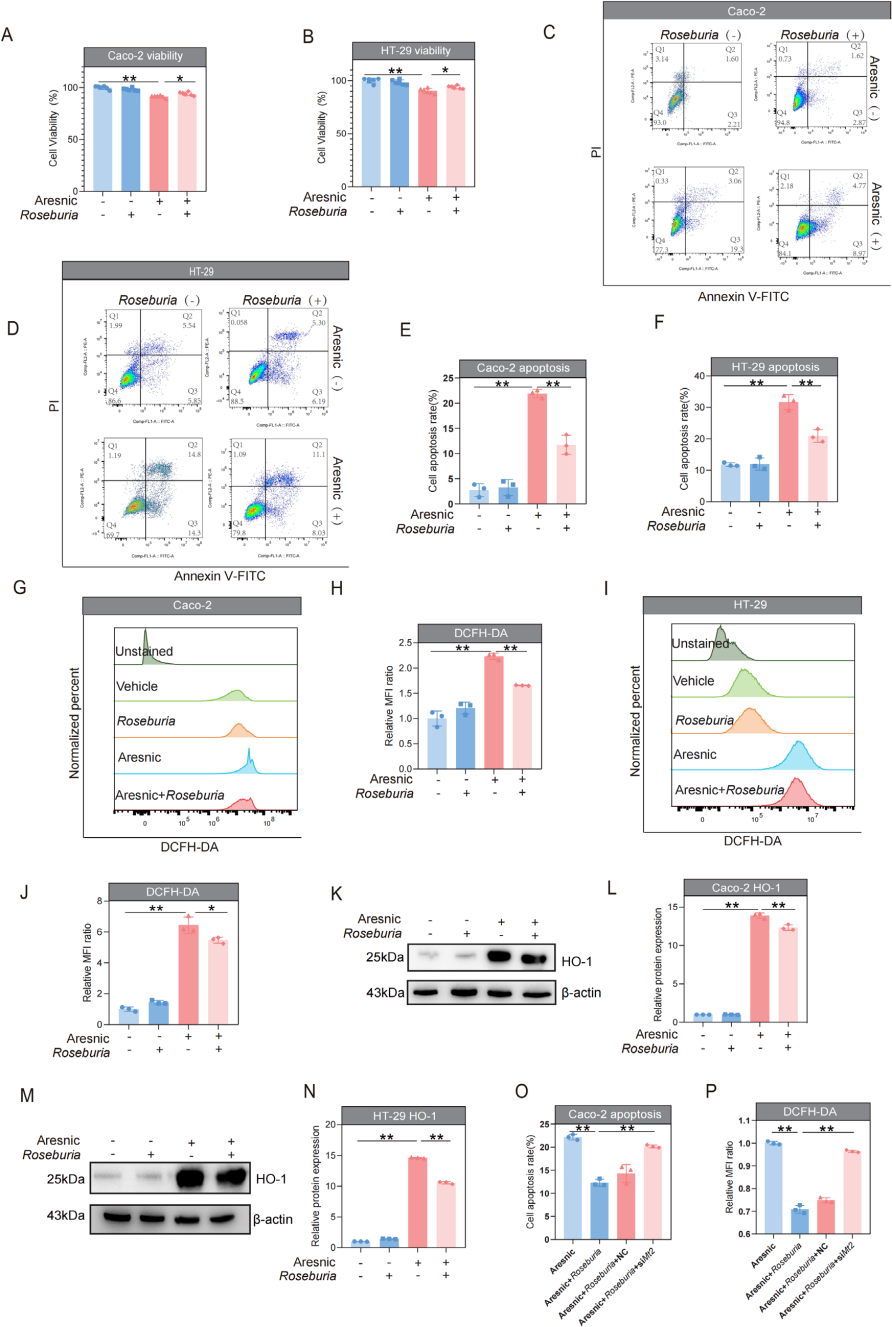
The culture supernatant of *Roseburia intestinalis* alleviated the toxic effects of arsenic on Caco‐2 and HT‐29 intestinal cell lines. A,B) The cell viability of Caco‐2 and HT‐29 cells after arsenic treatment with or without intervention with the culture supernatant of *Roseburia intestinalis* (*n* = 6). C–F) The representative flow cytometry analysis of apoptosis using Annexin V‐PI staining, and the corresponding apoptosis results for Caco‐2 and HT‐29 cells treated with arsenic, with or without intervention using *Roseburia intestinalis* culture supernatant (*n* = 3). G–J) The representative flow cytometry analysis of intracellular ROS using DCFH‐DA staining, and the corresponding ROS results for Caco‐2 and HT‐29 cells treated with arsenic, with or without intervention using *Roseburia intestinalis* culture supernatant (*n* = 3). K–N) The representative images of HO‐1 protein bands and corresponding quantitative analysis results for Caco‐2 and HT‐29 cells treated with arsenic, with or without intervention using *Roseburia intestinalis* culture supernatant (*n* = 3). O,P) The quantitative analysis of cellular apoptosis and intracellular ROS in Caco‐2 cells treated with arsenic plus *Roseburia intestinalis* culture supernatant intervention, with or without *Mt2* knockdown (*n* = 3). Data are expressed as mean ± S.D. ^*^ *p *< 0.05, ^**^ *p* < 0.01.

As demonstrated by the results of the cell viability and apoptosis assays, treatment with the culture supernatant of *Roseburia intestinalis* alone did not affect cell viability or apoptosis in either cell line or the control. Compared with cells treatment with arsenic alone, intervention with the culture supernatant of *Roseburia intestinalis* significantly suppressed the arsenic‐induced reductions in cell viability and increases in apoptosis in both the Caco‐2 and HT‐29 lines (**Figure** **10**A–F). Furthermore, the intracellular ROS levels increased more than 2‐fold in the arsenic‐exposed cells of both lines, whereas the culture supernatant of *Roseburia intestinalis*‐treated cells reduced arsenic‐induced ROS accumulation (Figure 10G–J). Notably, this ROS mitigation effect correlated with HO‐1 expression: arsenic alone triggerred markedly upregulated HO‐1 expression, while intervention with the *Roseburia intestinalis* culture supernatant suppressed the increase in HO‐1 expression, suggesting alleviation of oxidative stress (Figure 10K–N).

To further validate the role of *Mt2* identified through transcriptomics, we first measured *Mt2* expression in Caco‐2 cells under arsenic treatment with or without the *Roseburia intestinalis* culture supernatant intervention. Specifically, arsenic alone triggerred the downregulation of *Mt2* expression, whereas intervention with the *Roseburia intestinalis* culture supernatant suppressed the decrease in *Mt2* expression, suggesting alleviated cellular stress (Figure S7, Supporting Information). The *Mt2* gene was subsequently knocked down by siRNA in Caco‐2 cells, and the efficacy of siMt2 was verified, as shown in Figure S8 (Supporting Information). These results revealed that the culture supernatant of *Roseburia intestinalis* significantly suppressed the arsenic‐induced reduction in cell apoptosis and reduced arsenic‐induced ROS accumulation in Caco‐2 cells, whereas *Mt2* knockdown attenuated these protective effects (Figure 10O,P), further indicating the essential role of *Mt2* in mediating the alleviation of arsenic toxicity by *Roseburia intestinalis*.

## 3 Discussion

Increasing evidence has shown that exposure to arsenic, either chronic or acute, leads to the disturbances in the gut microbiome ^[^ ^61^, ^62^ ^]^. More importantly, in the gut environment, the microbiome profoundly affects arsenic bioaccumulation, transport, and even detoxication ^[^ ^63^, ^64^ ^]^. Therefore, the interaction between arsenic and gut microbiome has recently been regarded as an important foundational topic in the field of environmental toxicology, but we still need to understand the functions and reactions of the gut microbiome at a more detailed level. For example, in one recent report, FMT treatment with healthy maternal fecal microbiota was able to alleviate prenatal inflammation and intestinal barrier disruption induced by arsenic.^[^ ^65^ ^]^ However, very little information provides even the most rudimentary understanding of which key bioactive bacteria and/or metabolites affect the toxicity of arsenic in the intestine. To bridge this gap, in the present study, we identified a new role of a gut‐derived strain, *Roseburia intestinalis*, which has protective effects against arsenic‐induced intestinal barrier injury and changes in the gut microenvironment in mice. These findings provide direct experimental evidence for the use of this species for the treatment and prevention of arsenic toxicity.

In most mammals, the intestinal barrier is composed of epithelial cells connected by cell–cell junctions. This barrier can segregate host tissue and commensal bacteria and is crucial for maintaining tissue homeostasis.^[^ ^66^ ^]^ Breaking or even slightly perturbing the intestinal barrier can result in severe pathological consequences.^[^ ^67^ ^]^ In this study, we systemically assessed the influences of arsenic on intestinal barriers from mechanical, chemical, immunological, and biological aspects. Together, these experimental findings suggest that short‐term exposure to relatively high levels of arsenic severely damages intestinal tissue integrity and disturbs the gut microenvironment. The function of the epithelial barrier in the intestine is therefore obviously affected by arsenic. Additionally, defects in the intestinal barrier caused by arsenic can further promote the progression of various gastrointestinal diseases, such as functional gastrointestinal disease and inflammatory bowel disease.^[^ ^68^, ^69^ ^]^ Notably, our previous work and other reports have together conclusively shown that a healthy microbiome plays a crucial role in protecting the host from arsenic toxicity, but establishing a direct correlation between specific microbiome activities and specific outcomes is very difficult (^[^ ^64^, ^70^ ^]^). In this study, we confirmed that FMT treatment with healthy fecal microbiota could significantly alleviate arsenic‐induced disruption of different functions of the intestinal barrier. Importantly, we also employed high‐throughput sequencing of the 16S rRNA gene combined with anaerobic culture technology to identify and cultivate a bacterial strain, *Roseburia intestinalis*. Our results indicate that this strain effectively mitigated intestinal barrier damage caused by arsenic.

FMT has been proven to be an effective treatment for various diseases by restoring the diversity and function of the gut microbiome.^[^ ^71^ ^]^ One previous study revealed that transplantation of the human microbiome protected germ‐free *As3mt* knockout mice from arsenic‐induced mortality, and further evidence indicated that this beneficial effect depends on the stability of the gut microbiota and the presence of specific bacteria, such as *Faecalibacterium*.^[^ ^72^ ^]^ Another study conducted by Chen et al. revealed that the administration of healthy gut microbiota enhanced the excretion and biotransformation of arsenic and alleviated metabolic disorders in their mammalian host.^[^ ^27^ ^]^ Similar results were reported by Luo et al., in which the in situ arsenic‐induced fecal microbiome was shown to be stably colonized and interact with indigenous microbes in recipient mice, leading to lower plasma arsenic accumulation and fecal arsenic excretion.^[^ ^61^ ^]^ In this study, we found that altering the microbiome by FMT with healthy fecal microbiota had protective effects against arsenic toxicity, suggesting that the shift in the gut microbiome induced by arsenic may be the cause of many pathologies.

*Roseburia intestinalis*, a gram‐positive bacterium belonging to the phylum *Firmicutes*, is a widely colonized anaerobic species in the intestine. It is one of the top 20 most abundant bacteria in the gut and typically comprises 0.9%–5.0% of the total microbiota.^[^ ^73^ ^]^ The role of *Roseburia intestinalis* in modulating gut microbial ecology, immune responses, and the development of human disorders has gained much attention ^[^ ^74^ ^]^^[^ ^47^ ^]^). It can also be applied as a potential probiotic because of its ability to produce butyrate, which is a major type of SCFA.^[^ ^75^ ^]^ Recently, *Roseburia intestinalis* has been brought to the fore because of its novel role in sustaining the intestinal barrier and modulating the gut microbial ecology.^[^ ^73^ ^]^ Herein, we observed that exposure to arsenic caused a reduced abundance of *Roseburia intestinalis*. *Roseburia intestinalis* usually penetrates the mucus layer to interact with the epithelium, adhere to intestinal mucin, and thereby facilitate its beneficial effects as a probiotic.^[^ ^73^ ^]^ This decreased abundance of *Roseburia intestinalis* may directly lead to the loss of function of the dominant strains in the gut microbiome. Moreover, the severe damage to the intestinal barrier caused by arsenic may further aggravate the loss of ability of *Roseburia intestinalis* to maintain the normal gut microbial ecology. In this study, we also observed a very interesting phenomenon in which the administration of FMT did not significantly affect on the activity of maltase, although arsenic exposure led to a decrease in the level of its activity. Intriguingly, treatment with *Roseburia intestinalis* profoundly alleviated the reduction in maltase activity induced by arsenic. We speculated that the protective effects of *Roseburia intestinalis* might be achieved at relatively high concentrations. Notably, several external factors may affect the function of *Roseburia intestinalis*, such as diet and antibiotic use, and the protective effects of this species against arsenic toxicity may depend on these factors. The results of correlation analysis, revealed that 3‐*O*‐acetylepisamarcandin, PC(20:3/0:0), and formiminoglutaminc acid were positively correlated with the abundance of *Roseburia intestinalis*, whereas 4‐guanidinobutanoic acid and l‐proline were negatively correlated with its abundance. As a hydrolysis product of phospholipase A2, PC(20:3/0:0) (lysophosphatidylcholine) can inhibit the activation of NKT cells and reduce the release of IL‐6/TNF‐α at physiological concentrations. However, when the microbiota is imbalanced, it is further hydrolyzed by bacterial phospholipase into lysophosphatidic acid (LPA), which promotes macrophage infiltration and IL‐1β secretion and directly damages the intestinal epithelial barrier.^[^ ^76^, ^77^, ^78^ ^]^ 4‐Guanidinobutanoic acid (4‐GBA) is a metabolite of arginine that can activate the cGAS‐STING/NLRP3 inflammasome when it accumulates in inflammatory bowel disease.^[^ ^79^ ^]^ The correlations between *Roseburia bacteria* and lysophosphatidylcholine as well as 4‐GBA suggest that it may be involved in the intestinal damage caused by arsenic exposure through inflammation‐related mechanisms. However, how *Roseburia bacteria* specifically affect the expression of these two metabolites remains to be further studied. The metabolite formiminoglutamic acid (FIGLU) is considered a marker of folic acid deficiency, however, folic acid deficiency can inhibit the synthesis and repair of intestinal epithelial DNA and delay ulcer healing,^[^ ^80^ ^]^ whereas exogenous proline has been shown to activate intestinal lymphoid tissue‐inducer (LTi) cells, thereby promoting intestinal homeostasis and relieving colitis.^[^ ^81^ ^]^ However, there is currently a lack of direct research evidence on how *Roseburia bacteria* affect the contents of FIGLU and L‐proline contents.

Through transcriptomic analysis and in vitro experiments, we herein demonstrated that the administration of *Roseburia intestinalis* possibly attenuates arsenic toxicity in the intestine by antagonizing oxidative stress and reducing apoptosis. Genes such as *Igfbp5*, *Krt5*, and *Mt2*, which play important roles in regulating cell proliferation, maintaining structural stability, and defending against oxidative stress, may be involved in the functional mechanisms. Combined with the metabolomic data and previous reports, the altered metabolite 4‐GBA can induce NLRP3 inflammasome activation,^[^ ^79^ ^]^ while the latter one is a potent inhibitor of *Mt2* transcription via JAK/STAT signaling.^[^ ^82^, ^83^ ^]^ These findings suggest that the metabolite 4‐GBA may play an important role in mediating the impact of arsenic exposure on the gut and the protective effects of *Roseburia intestinalis*, possibly by regulating the expression of *Mt2*, which is critical for maintaining intestinal integrity.^[^ ^84^ ^]^ What noteworthy is, *Roseburia intestinalis* most likely exerts its protective effects against arsenic‐induced gut injury not in isolation, but through dynamic interactions with the commensal microbiota and the host microenvironment. Previous reports indicate that *Roseburia intestinalis* produces butyrate by degrading complex polysaccharides, which are further metabolized by commensal bacteria such as *Faecalibacterium*, forming a cross‐feeding chain.^[^ ^74^, ^85^ ^]^ Moreover, butyrate secreted by *Roseburia intestinalis* enhances intestinal hypoxia, promoting the colonization of strict anaerobes such as *Lachnospiraceae*, while suppressing facultative anaerobic pathogens such as *Enterobacteriaceae*.^[^ ^86^ ^]^ Additionally, *Roseburia intestinalis*‐derived butyrate activates the PPARγ pathway in goblet cells, stimulating Muc2 secretion.^[^ ^87^ ^]^ The thickened mucus layer in turn provides an ecological niche for *Roseburia intestinalis* and other mucophilic symbionts, establishing a self‐reinforcing“mucus restoration‐microbiota colonization”positive feedback loop.

There are four major limitations in this study that should be addressed. First, the metabolites associated with *Roseburia intestinalis* were screened and identified by correlation analysis, and we could not confirm the causal link between *Roseburia intestinalis* and the related metabolites in this way. Second, the arsenic dosage used herein seemed relatively higher than that present in the natural environment because of the consideration about uncertainty factor for animal‐to‐human extrapolation and acute or subacute arsenic exposure, which occurs in cases of drug abuse or arsenic‐contaminated drinking water poisoning. Third, the duration of arsenic exposure was relatively short, therefore, we could not determine whether the protective effects of *Roseburia intestinalis* against acute arsenic toxicity would be maintained in a long‐term exposure model. Fourth, although we conducted the transcriptomics for the screening of potential target genes and related pathways involved in the beneficial effects of *Roseburia intestinalis*, its molecular mode of action remains currently unknown.

In summary, in this study, we revealed that acute exposure to arsenic caused defects in the mechanical, chemical, immunological, and biological barriers of the intestine, thereby changing the microenvironment in the gut. By administrating FMT with healthy fecal microbiota, we verified that restoring the gut microecology alleviated the intestinal damage induced by arsenic. More importantly, we established for the first time that a novel gut‐derived strain, *Roseburia intestinalis*, exhibited significant protection against arsenic‐induced intestinal barrier disruption and alteration of the gut microenvironment. These findings not only provide a new insight for identifying interventions or uncovering mechanisms from the perspective of the gut microbiome, but also imply that supplementation with gut‐derived bacteria is an alternative strategy for the prevention and treatment of arsenic‐related intestinal disorders.

## 4 Experimental Section

### Animals and Treatment

Healthy C57BL/6J male mice with 8 weeks of age and 18–22 g of weight were purchased from the Experimental Animal Center of Chongqing Medical University (Animal certificate number: Chongqing, SCXK (Yu) 2022‐0016). Mice were kept under a controlled condition with the temperature at 23 ± 1 °C, relative humidity of 50% ± 10%, and a light–dark cycle of 12/12 h. Before the experiment, they were acclimatized to the environment for 1 week, and all the animals were free access to food and water. For the arsenic exposure protocol, mice were randomly divided into 3 groups by using website https://www.randomizer.org, with at least 10 mice in each group. The expected effect size was selected based on the “3 R” principles (Replacement, Reduction, and Refinement) and the sample size was estimated by the G Power program. In detail, for an experiment with three treatment groups with an effect size of 0.9, a power of 0.8 and a significance level of 0.05, 29 animals would be required, with each group containing about 10 animals. In the arsenic‐treated groups, the mice were fed with 50 mg L^−1^ arsenic (the low‐dose group) or 100 mg L^−1^ arsenic (the high‐dose group, arsenic purity ≥ 90%, Sigma Aldrich Chemical Co., Cat#S7400, St. Louis, MO, USA, dissolved in sterile water) via drinking water for 2 weeks, while mice in the vehicle group were treated with sterile water. The arsenic doses were selected mainly based on the following reasons: First, a range of 0.05−3.2 mg L^−1^ arsenic level was reported in high arsenic contaminated groundwater,^[^ ^88^ ^]^ given the uncertainty factor between human and mouse (2‐ to 10‐folds), a exposure dose of 50 mg L^−1^ was set. Second, in cases of acute arsenic poisoning, the arsenic ingested doses usually ranges from 5 to 130 mg kg^−1^ day^−1^,^[^ ^4^, ^89^ ^]^ the doses used here were within the doses (equal to 10–40 mg kg^−1^ day^−1^). Third, similar doses have been used in previous studies to study the acute and sub‐acute toxic effects of arsenic.^[^ ^90^, ^91^, ^92^ ^]^ During the treatment, the arsenic solution was prepared freshly every 2 days. All experimental procedures involving animals in the study were approved by the Chongqing Medical University Institutional Animal Care and Use Committee (Approved Number: Yu‐2022‐0016), and every effort was made to reduce animal suffering. At the end of administration, the feces were collected. Then, mice were sacrificed under anesthesia, and the blood and ileum of mice were collected for further designed experiments.

### Fecal Microbiota Transplantation

FMT was performed according to previous established protocols with minor modifications.^[^ ^93^ ^]^ In line with animals used in the above‐mentioned arsenic treatment, 30 male mice were randomly divided to control group, arsenic group and arsenic + FMT group, with 10 mice in each group, respectively. To improve the engraftment of donor microbiota, all the mice were pretreated with antibiotics prior to FMT. An antibiotics cocktail (combination of 100 mg kg^−1^ ampicillin, 100 mg kg^−1^ neomycin, 100 mg kg^−1^ metronidazole, 50 mg kg^−1^ vancomycin, and 1 mg kg^−1^ amphotericin B) was administrated to the animal by intragastric gavage twice a day for a week. After antibiotics administration, the mice in the arsenic and arsenic + FMT groups were fed with 50 mg L^−1^ arsenic via drinking water for additional 2 weeks, the mice in control group were fed with sterile water. For the FMT protocol, the microbiota was obtained from feces of untreated healthy mice. In brief, fecal samples used for FMT were freshly collected from healthy mice and suspended with sterile phosphate‐buffered saline (PBS) (20 mg feces mL^−1^). After vortexing, the fecal suspension was centrifuged and the supernatant was collected and mixed with glycerol at the ratio of 5:1. During the treatment of arsenic, the animals in the arsenic + FMT group were intragastric gavage daily with 200 µL of mixed solution from supernatant. The mice in the control group and arsenic group received equivoluminal sterile PBS which containing 20% glycerol.

### Culture of *Roseburia Intestinalis* and Treatment

*Roseburia intestinalis* L1‐82 (DSM14610) was obtained from Deutsche Sammlung von Mikroorganismen und Zellkulturen GmbH (Braunschweig, Germany) and was cultured at 37 °C in reinforced clostridial medium in an anaerobic culture environment created by using an E200G anaerobic chamber (Chongqing Jiangxue Science & Technology Ltd., Chongqing, China).The viability of Laboratory‐Cultured *Roseburia intestinalis* was analyzed using fluorescence staining (presented in the Supporting Information file). As for the *Roseburia intestinalis* intervention experiment design, the expected effect size was also selected based on the “3 R” principles and the sample size was estimated by the G Power program using the same parameter settings as mentioned in the arsenic exposure experiment. Based on the calculation result, the minimum required sample size was 29. To use a round number, 32 mice were ultimately employed, and these mice were randomly divided to four groups (control group, *Roseburia intestinalis* group, arsenic group, arsenic+ *Roseburia intestinalis* group) with 8 mice in each group. Mice in the arsenic group and arsenic + *Roseburia intestinalis* group were fed with arsenic via drinking water as mentioned above. Before transplantation, the bacterial cells were collected by centrifuging at 8000 *g* for 10 min, washed with sterile PBS, and finally resuspended with PBS at a concentration of 10^9^ CFUs mL^−1^. Each mouse in the *Roseburia intestinalis* group and arsenic + *Roseburia intestinalis* group was administrated intragastrically with 200 µL of *Roseburia intestinalis* suspension daily for 14 days. The abundance of *Roseburia intestinalis* in feces of mice was determined by extracting fecal genomic DNA from mouse stools and quantified by qPCR. The corresponding methods were presented in the Supporting Information Methods.

### Measurement of Sucrase, Lactase, and Maltase Activities

The sucrase, lactase, and maltase activities in the ileum were analyzed by using commercial assay kits (Nanjing Jiancheng Bioengineering Institute, Cat#A082‐1‐1, Cat#A082‐2‐1, Cat#A082‐3‐1, Nanjing, China) following the manufacturer's instructions. In brief, the tissues were homogenized in PBS, and the supernatant was collected after centrifugation at 2500 rpm for 10 min. The supernatant was then used for the measurement of activities. The absorbance was detected by a Varioskan LUX multimode microplate reader (VLBL0TD2, Thermo fisher scientific, MA, USA) at the wavelength of 505 nm, and the activity was normalized by the corresponding protein concentrations determined by enhanced Bicinchoninic acid (BCA) Protein Quantification Kit (Beyotime, Cat# P0009, Shanghai, China).

### Histological Analysis

The intestinal tissues in each group were removed immediately after the mice being euthanized, and then fixed with 4% paraformaldehyde, dehydrated in ethanol, embedded in paraffin and cut into 5 µm sections. To assess the histological changes and compare the number of goblet cell as well as mast cell, the prepared sections were dewaxed with xylene and hydrated, and stained with H&E (Solarbio Biotech Co., Ltd., Cat#G1121, Beijing, China), AB‐PAS (Solarbio Biotech Co., Ltd., Cat#G1285, Beijing, China), and TBO (Solarbio Biotech Co., Ltd., Cat#G3661, Beijing, China). After sealing with neutral resin, the sections were observed with an Olympus optical microscope (BX53F2, Tokyo, Japan). The histopathological scoring was carried out according to previously defined criteria including five categories: inflammation, epithelial damage, edema, goblet cell loss, and mucosal hyperplasia.^[^ ^94^ ^]^ The number of goblet cell and mast cell were calculated by using ImageJ software (version 1.37, National Institutes of Health, MD, USA).

### Immunohistochemistry Assay

Immunohistochemistry assay was performed according to the protocols described previously.^[^ ^95^ ^]^ In brief, the paraffin sections were dewaxed with xylene and dehydrated with alcohol. Afterwards, heat induced antigen retrieval was carried out using sodium citrate. Then, the sections were quenched with fresh 3% hydrogen peroxide to inhibit endogenous peroxidase activity. After incubating with nonimmune goat serum, the sections were subsequently incubated with anti‐lysozyme primary antibody (1:1000) at 4 °C overnight, followed by incubation with biotinylated secondary antibody. Thereafter, the sections were treated with color developing agent and hematoxylin. After mounting with neutral resins, the sections were observed using an Olympus optical microscope (BX53F2, Tokyo, Japan). The images were analyzed and the integrated option density was calculated using ImageJ software (version 1.37, National Institutes of Health, MD, USA).

### Immunofluorescence Assay

Ileum samples were fixed in 4% PFA, dehydrated in a serious of sucrose gradient, and frozen in OCT medium. Then, 7–8 µm sections were prepared, blocked with 4% BSA solution, and subjected to Muc2 (1:500, Proteintech, Cat#27675‐1‐AP, Wuhan, China) staining at 4 °C overnight. After thorough washing with PBST, Alexa Fluor 488‐conjugated chicken anti‐rabbit IgG (H+L) cross‐adsorbed secondary antibody served as a secondary antibody (1:100, Invitrogen, Cat#A‐21441, Carsbad, CA, USA) and incubated the tissue sections at room temperature for 1 h, followed by washing with PBST. Thereafter, sections were incubated with DAPI (ThermoFisher, Cat#A2179275, MA, USA), mounted with antifade mounting medium and the images were captured using a Nikon Eclipse C1 fluorescence microscope (Tokyo, Japan).

### 16S rRNA Gene Sequencing

The 16S rRNA gene sequencing was conducted as previously reported.^[^ ^96^ ^]^ In brief, the fresh fecal samples were collected from twelve mice in each group. Then, the total microbial genomic DNA was extracted using PF Mag‐Bind Stool DNA Kit (Omega Bio‐Tek, Cat#M9636‐02GA, USA), and PCR amplification of the hypervariable region V3‐V4 of the bacterial 16S rRNA gene were performed using the upstream primer pairs (338F: 5′‐ACTCCTACGGGAGGCAGCAG‐3′, 806R: 5′‐GGACTACHVGGGTWTCTAAT‐3′) by an ABI GeneAmp9700 PCR thermocycler (ABI, CA, USA). Purified PCR products were pooled in equimolar amounts and paired‐end sequenced on an Illumina PE300 platform (Illumina, San Diego, USA). Fastp (version 0.19.6), FLASH (version 1.2.11), and UPARSE (version 11) softwares were used for data pre‐processing. Species annotation and abundance analysis were carried out with 16S rRNA gene reference database Silva v138.1. The statistical analysis was conducted mainly by using Mothur (version 1.30.2) software and Vegan v2.4.3 package of R language (version 3.3.1). Specifically, α‐diversity was assessed using the metrics including Observed species (Sobs), Simpson, Shannon, Chao, Ace and the coverage diversity indexes. As for β‐diversity, PCA and PLS‐DA were used to reflect differences in species complexity and the separation between groups. Furthermore, the profile differences in microbial community abundance and the significantly altered microbial were assessed to investigate the component characteristics and specific microbial changed by arsenic.

### Non‐targeted Metabolomics and Analysis

Non‐targeted metabolomics was applied to assess the changes of metabolites in plasma of mice after indicated treatment. Briefly, the collected blood was centrifuged at 3000 rpm for 10 min to obtain serum, then, 100 µL serum was added to a 1.5 mL centrifuge tube with 400 µL solution^[^acetonitrile: methanol = 1:1(v:v)] containing 0.02 mg mL^−1^ internal standard (L‐2‐chlorophenylalanine) to extract metabolites. Thereafter, the sample matrix was mixed by vortexing for 30 s, followed by sonication in an ice‐water bath for metabolites extraction. Then, the samples were centrifuged at 4000 *g* for 15 min at 4 °C after precipitating proteins at −20 °C. The supernatant was blown dry with nitrogen, then residues were redissolved with 100 µL solution (acetonitrile: water = 1:1) and ultrasonicated for 5 min (5 °C, 40 kHz). After centrifugation at 13 000 *g* and 4 °C for 10 min, the supernatant was collected and analyzed using liquid chromatography‐tandem mass spectrometry (LC‐MS/MS). Quality control was performed using a pooled quality control sample (QC) by mixing equal volumes of all samples. Ten microliter of supernatant was mixed into QC samples and injected for testing with a Thermo UHPLC‐Q Exactive HF‐X system equipped with an ACQUITY HSS T3 column (100 × 2.1 mm i.d., 1.8 µm; Waters, USA) at Majorbio Bio‐Pharm Technology Co. Ltd. (Shanghai, China). The mobile phases consisted of buffer A (0.1% formic acid in water and acetonitrile with a proportion of 95:5 in volume) and buffer B (0.1% formic acid in acetonitrile and isopropanol:water with a proportion of 47.5:47.5 in volume). The flow rate was set at 0.40 mL min^−1^, while the column temperature was set at 40 °C. The mass spectrometric data was collected with a Thermo UHPLC‐Q Exactive HF‐X Mass Spectrometer coupled to an electrospray ionization (ESI) was operated in positive/negative polarity modes.

LC/MS raw data was imported into Progenesis QI (Waters Corporation, Milford, USA) software for pre‐processing. Then, data was analyzed using the free online platform of Majorbio Cloud Platform at the website of https://cloud.majorbio.com. PCA and orthogonal least partial squares discriminant analysis (OPLS‐DA) were performed with the R package “ropls” (Version 1.6.2). The differential metabolites between groups were recognized based on the Variable importance in the projection (VIP) and the *p*‐value generated by Student's *t* test as the following criteria: VIP scores ≥1, *p* < 0.05. Both volcano plots and heatmaps were used to visualize the differences in metabolite levels. To further investigate the metabolite functions, metabolic enrichment and pathway analysis were carried out based on the Kyoto Encyclopedia of Genes and Genomes (KEGG, http://www.genome.jp/kegg) database.

### Cell Culture and Treatment

The Caco‐2 cells were derived from human colon cancer cells and could differentiate into absorptive enterocytes during culture, effectively simulating the transport and absorption processes of human small intestinal epithelial cells. While the HT‐29 cells originate from the primary tumor of a white female with colorectal cancer and serve as a unique model for studying the molecular mechanisms of intestinal cell differentiation. Both cell lines were applied to elucidate the potential mechanism of how *Roseburia intestinalis* exerts its protective effect.

The Caco‐2 cells and the HT‐29 cells used in this study were purchased from the ATCC Cell Bank (Immocell Biotechnology Co., Ltd., Xiamen, China, Cat: IM‐H092, RRID: CVCL_0025 and Cat: IM‐H102, RRID: CVCL_0320, respectively). The Caco‐2 cells were cultured in MEM Eagle's medium (Hyclone, Logan, UT) supplemented with 20% FBS and 1% penicillin‐streptomycin, while the HT‐29 cells were cultured in McCoy's 5A medium (Hyclone, Logan, UT) supplemented with 10% FBS and 1% penicillin‐streptomycin. Both of the cell lines were cultured in an incubator with 5% CO_2_ at 37 °C, passaging was performed when the cell confluence reached 80%–90%.

The Caco‐2 cells and the HT‐29 cells seeded on 6‐well plates were divided into four groups, respectively, means the control group, the arsenic group, the *Roseburia intestinalis* group, and the arsenic + *Roseburia intestinalis* group. The control group was treated with cell culture medium; the arsenic group was treated with 5  µmol sodium arsenic, while the arsenic dose selected based on results of CCK8 assay (different arsenic concentrations were tested and the results were presented in the Supporting Information file); the *Roseburia intestinalis* group was treated with the *Roseburia intestinalis* culture supernatant. Indeed, dose‐response optimization assays were performed to identify the optimal dose (the detailed methods and results were presented in the Supporting Information file) and finally found the strongest protective effect was observed at a 1:1 dilution (v/v) of *Roseburia intestinalis* culture supernatant with fresh cell medium. Specifically, the *Roseburia intestinalis* culture supernatant was prepared as follows: *Roseburia intestinalis* were cultured in anaerobic broth to the late‐logarithmic phase, then they were harvested by centrifugation (8000 × *g*, 4 °C, 10 min), washed twice with sterile PBS, and adjusted to OD600 = 1.0. Antibiotic‐free cell culture medium was subjected to oxygen depletion in an anaerobic chamber, after which 1 mL of the standardized bacterial suspension was aseptically added to 50 mL cell medium and cocultured in a cell culture incubator (37 °C, 5% CO_2_) for 24 h. After that, the supernatant was collected (8000 × *g*, 4 °C, 10 min), filter‐sterilized with 0.22 µm membrane filter, and stored at −80 °C. Prior for cell treatment in the *Roseburia intestinalis* intervention group, the above‐mentioned supernatant was mixed 1:1 with fresh cell medium. As for the arsenic + *Roseburia intestinalis* group, cells were treated with a 1:1 mixture of *Roseburia intestinalis* culture supernatant and fresh culture medium, supplemented with sodium arsenite to a final concentration of 5 × 10^−6^ m. Cell viability, intracellular ROS levels, cell apoptosis rates, and protein content were measured after 24 h of treatment, the detailed methods were described in the Supporting Information section.

To validate the role of the key genes identified through transcriptomics, gene knockdown was performed by siRNA and cell apoptosis and intracellular ROS accumulation was measured in Caco‐2 cells, and the corresponding methods were provided in the Supporting Information file.

### Quantitative PCR (qPCR) Assay

The mRNA expressions of *occludin*, tight junction protein‐1 (*Tjp‐1)*, interleukin‐1 beta (*Il‐1β*), interleukin‐6 (*Il‐6*), tumor necrosis factor α (*TNF‐α*), and T cell‐associated transcription factor (*T‐bet*), insulin like growth factor binding protein 5 (*Igfbp5*), keratin 5 (*Krt5*), and metallothionein 2 (*Mt2*) were detected by quantitative reverse‐transcriptase polymerase chain reaction (qPCR) assay. Total RNA was extracted using Eastep Super Total RNA extraction kit (Promega Co., WI, USA). Then, cDNA was reverse transcribed using a TransStart Tip Green qPCR SuperMix (TransGen Biotech, Cat#G891‐1, Beijing, China) and reacted with specific primers using a FastStart Universal SYBR Green Master Mix (ROX) (Roche, Switzerland, Basel). Primers used in this work were synthesized by Sangon Biotechnology Co., Ltd. (Shanghai, China) and sequences were shown in Table S1 (Supporting Information). Beta‐actin were served as a loading control. The relative expressions of mRNA were quantified with the 2‐^△△Ct^ method.

### Western Blot Analysis

The total proteins from the tissues or cultured cells were extracted using radio‐immunoprecipitation assay (RIPA) buffer (Beyotime, Cat#P0013B, Shanghai, China) containing protease inhibitor cocktail (ThermoFisher, Cat#A32955, MA, USA) and the protein concentrations were determined with the BCA protein assay kit (Beyotime, Cat#P0009, Shanghai, China). Proteins were denatured by heating and separated by sodium dodecyl sulphate‐polyacrylamide gel electrophoresis (SDS‐PAGE). After being transferred to polyvinylidene fluoride (PVDF) membrane and blocked with 5% non‐fat milk, the membranes were incubated with primary antibodies including Occludin (1:10000, Proteintech, Cat#27260‐1‐AP, Wuhan, China), ZO‐1 (1;1000, Affinity Biosciences, Cat#AF5145, Jiangsu, China), HO‐1 (1:3000, Proteintech, Cat#10701‐1‐AP, Wuhan, China), and β‐actin (1:10000, Abclonal, Cat#AC026, Wuhan, China). The PVDF membranes were then washed with TBST and incubated with a secondary antibody (1:100000, EarthOx, Cat#E030120‐01, CA, USA), followed by chemiluminescence detection using a ChemiDoc Touch Imaging System (BioRad, CA, USA). The protein bands were quantified by densitometry using ImageJ software (version 1.37, National Institutes of Health, MD, USA) and the relative protein expression levels were normalized to the β‐actin.

### Transcriptomic Analysis

To further explore how Roseburia exerted the protective effect against asenite‐induced ileal damage, transcriptomic analysis was applied to determine the expression of genes in ileum. Briefly, ileum of mice in the control group, R.i group and arsenic+ R.i group were collected for transcriptomic analysis which carried out with the same general method as previous reports.^[^ ^97^ ^]^ Briefly, total RNA of the ileum tissues was extracted using Trizol Reagent (Invitrogen) based on the manufacturer's instructions. Then the quality and quantity of the extracted RNA were analyzed with the aid of Bioanalyzer 2100 system (Agilent Technologies, CA, USA) and ND‐2000 (NanoDropTechnologies, Wilmington, USA). RNA samples with high quality (total RNA ≥ 1 µg, concentration ≥ 35 ng µL^−1^, OD260/280 ≥ 1.8, OD260/230 ≥ 1.0) were applied for cDNA generation and sequencing library construction, which were accomplished by Majorbio Bio‐pharm Biotechnology Co., Ltd. using a Illumina HiSeq X10 (Illumina).

Raw data was processed by SeqPrep (https://github.com/jstjohn/SeqPrep) and Sickle (https://github.com/najoshi/sickle) to obtain high‐quality clean data. After that, the clean reads were mapped to the reference genome by using the TopHat (http://tophat.cbcb.umd.edu/, version2.1.1) software. And then FPKM of each gene was calculated according to the length of the gene and reads count, followed by quantifying the gene abundances using RSEM (http://deweylab.biostat.wisc. edu/rsem/. Finally, the differentially expressed genes were identified using a R statistical package software DESeq2 (http://bioconductor.org/packages/stats/bioc/DESeq2.html) (*p*‐value of 0.05 and absolute fold change of 2). The enriched KEGG pathway analysis was implemented by KOBAS 2.1.1 (http://kobas.cbi.pku.edu.cn/download.php).

### Statistical Analysis

Unless other stated in the gut microbiota and metabolome sections, GraphPad Prism 8.0 (GraphPad, Inc. La Jolla, CA, USA) was used for the statistical analyses and make visualizations. The results were indicated as mean ± standard deviation (S.D.). As for arsenic exposure model, one‐way analysis of variance (ANOVA) was carried out for comparison when data was normally distributed, otherwise, Kruskal–Wallis H test was performed to compare the significant difference among groups, followed by post hoc test by using Tukey test or Dunn's test for parametric samples and nonparametric samples, respectively. As for data analyses in the FMT model and *Roseburia intestinalis* treatment model, the significant difference between two groups was evaluated by two‐tailed unpaired Student's *t*‐test if data was parametric, and assessed by Mann–Whitney *U* test if data was nonparametric. Spearman's correlation test was performed to analyze the relationship between gut microbiota and serum metabolites. *p*‐value < 0.05, was considered statistically significant.

## Conflict of interest

The authors declare no conflict of interest.

## Author Contributions

L.Z., C.W., J.G., X.W., and G.L. contributed equally to this work. L.X.Z. performed methodology, investigation, software, data curation, validation, reviewed and edited the writing. W.C.S. performed methodology and formal analysis. J.Y.G. performed resources, validation, reviewed and edited the writing. X.W. and G.L. worked on methodology and formal analysis. X.J.J. worked on resources and funding acquisition. Y.Y.X. worked on data curation and J.Z. worked on resources and validation. B.L. worked on resources and validation. F.Z. and H.Y.Z. performed formal analysis; H.F.P. worked on resources and validation. J.F.Q. performed conceptualization, supervision, and funding acquisition. S.C.X. performed conceptualization, supervision, and funding acquisition. Z.Z. worked on project administration, conceptualization, funding acquisition, reviewed and edited the writing. C.Z.C. worked on project administration, conceptualization, funding acquisition, reviewed and edited the writing.

## Declarations—Ethics Approval and Consent to Participate

The Chongqing Medical University Institutional Animal Care and Use Committee approved all protocols (Approved Number: Yu‐2022‐0016). Furthermore, every effort was made to reduce animal suffering.

## Supporting information



Supporting Information

## Data Availability

The data that support the findings of this study are available from the corresponding author upon reasonable request.;
