# Supplementary material for: Novel Role of Gut‐Derived Roseburia Intestinalis in Safeguarding Intestinal Barrier Integrity and Microenvironment Homeostasis During Arsenic Exposure
Source: Adv Sci (Weinh). 2025 Aug 19;12(42):e11895. doi: 10.1002/advs.202511895 (PMC12622465; doi:10.1002/advs.202511895)
Supplement: Supplementary file 1 — Supporting Information [file ADVS-12-e11895-s001.docx]

***Supporting Methods***

***Viability analysis of Laboratory-Cultured Roseburia intestinalis***

*Roseburia intestinalis* cultures were harvested during the late-logarithmic growth phase. The bacterial suspension was centrifuged at 10,000 × g for 10 min at 4°C, then the supernatant was discarded and the pellet was re-suspended in 25 mL sterile PBS and incubated at room temperature for 1 h. After repeating centrifugation (10,000 × g, 4°C, 10 min), the *Roseburia intestinalis* cultures were washed and re-suspended in sterile PBS. Optical density at 600 nm was measured to determine the growth state of bacterial cultures using a spectrophotometer. Given the loss of linearity at OD600 values >1.0, the sample were diluted until the culture density reached an OD 600 of about 1.0. Thereafter, a total of 1 mL of adjusted bacterial suspension was mixed with SYTO 9 and propidium iodide (PI) (Maokang Bio Co., Ltd., Cat#: MX4234, Shanghai, China), incubated in the dark at room temperature for 15 min. Finally, about 5 μl of the stained suspension was pipetted onto a glass slide, covered with a coverslip, and visualized using a fluorescence microscope.

***Quantification of Roseburia intestinalis Abundance in Feces of mice***

The abundance of *Roseburia intestinalis* was determined by extracting fecal genomic DNA from mouse stools. Specifically, fresh fecal samples from mice (n=6/group) were flash-frozen in liquid nitrogen and stored at -80°C until processing. Genomic DNA was extracted using the Fecal Genomic DNA Extraction Kit (Tiangen Biotech Co, Ltd., Cat#: DP328, Beijing, China). Subsequently, DNA concentration was measured with a micro-spectrophotometer and normalized to 1000 ng/mL. QPCR targeting *Roseburia intestinalis* was conducted on a CFX Connect™ Real-Time System (Bio-Rad) using 10 μL reactions containing: 5 μL TB Green® Premix Ex Taq™ II (Tli RNaseH Plus, Takara Bio) , 0.25 μL each of specific primers (10 μM), 3.5 μL nuclease-free water, and 1 μL template DNA. The forward primer sequence for *Roseburia intestinalis* was:GTCGCATGACCTGGTGTG, the reverse primer sequence was:AGTCAGGTACCGTCATTTC. UNI 16S gene served as the internal reference (Forward Primer: 5’-ACTCCTACGGGAGGCAGCAGT-3’, Reverse Primer: 5’-ATTACCGCGGCTGCTGGC-3’). The 2^(-ΔΔCt) method was used to calculate relative changes in gene expression.

***Cell viability assay***

Cell viability was determined by the CCK8 assay. The Caco-2 cells and the HT-29 cells (1 × 10^4^ /well) were seeded in a 96-well plate overnight before treatment. For arsenic dose selection, cells were treated with different concentrations of arsenic (0, 0.1, 0.25, 0.5, 1, 2.5, 5, 10, 20 μM). For evaluation of *Roseburia intestinalis* intervation, cells in the control group, the arsenic group, the *Roseburia intestinalis* group, and the arsenic + *Roseburia intestinalis* group were treated with cell culture medium, 5 μmol sodium arsenic, a 1:1 mixture of *Roseburia intestinalis* culture supernatant and fresh culture medium, a 1:1 mixture of *Roseburia intestinalis* culture supernatant and fresh culture medium supplemented with 5 μmol sodium arsenic, respectively. After 24 h of treatment, ten microliter of CCK-8 reagent (Tongren Institute of Chemistry, Cat#: CK04, Shenyang, China) was added to each well, and the plate was incubated for an additional 2 h at 37 °C in the dark. Then, the absorbance of each sample at 450 nm and 600 nm (reference wavelength) were immediately evaluated using a VERSAmax Microplate Reader (Molecular Devices, Sunnyvale, CA, USA). Cell viability (%) = [A_treatment_ - A_blank_] / [A_control_ - A_blank_] × 100.

To identify the optimal dose of *Roseburia intestinalis* in the in vitro experiments, we performed dose-response optimization analysis using CCK8 assay. In detail, six groups were established: the control group (untreated cells), the arsenic group (treated with 5 μM sodium arsenite), and four co-treatment groups receiving arsenic plus serially diluted *Roseburia intestinalis* culture supernatant (at ratios of 2:1, 1:1, 1:2, and 1:4 v/v). Specifically, the *Roseburia intestinali*s culture supernatant was prepared as follows: *Roseburia intestinalis* were cultured in anaerobic broth to the late-logarithmic phase, then they were harvested by centrifugation (8,000 × g, 4°C, 10 min), washed twice with sterile PBS, and adjusted to OD600 = 1.0. Antibiotic-free cell culture medium was subjected to oxygen depletion in an anaerobic chamber, after which 1 mL of the standardized bacterial suspension was aseptically added to 50 mL cell medium and co-cultured in a cell culture incubator (37°C, 5% CO₂) for 24 hours. After that, the supernatant was collected (8,000 × g, 4°C, 10 min), filter-sterilized with 0.22 μm membrane filter, and stored at -80°C. Prior for cell treatment, the above-mentioned supernatant was mixed 2:1，1:1，1:2 and 1:4 with fresh cell medium. After treatment, the cell viability of Caco-2 cells were determined as above-described in the section for arsenic dose selection.

***Intracellular ROS and cell apoptosis determination***

The Caco-2 cells and the HT-29 cells were seeded in 12-well plates overnight, cells in the control group, the arsenic group, the *Roseburia intestinalis* group, and the arsenic + *Roseburia intestinalis* group were treated with cell culture medium, 5 μmol sodium arsenic, a 1:1 mixture of *Roseburia intestinalis* culture supernatant and fresh culture medium, a 1:1 mixture of *Roseburia intestinalis* culture supernatant and fresh culture medium supplemented with 5 μmol sodium arsenic, respectively. After 24 h of treatment, cells were trypsinized from the plate and collected for intracellular ROS and cell apoptosis determination.

To validate the role of the key genes identified through transcriptomics, gene knockdown was performed by siRNA and cell apoptosis and intracellular ROS accumulation was measured in Caco-2 cells co-treated with arsenic plus *Roseburia intestinalis* culture supernatant in the following four groups: arsenic exposure group, arsenic exposure + *Roseburia intestinalis* intervention group, arsenic exposure + *Roseburia intestinalis* + empty vector transfection group, and arsenic exposure + *Roseburia intestinalis*+ *Mt2*-knockdown group. The arsenic treatment and *Roseburia intestinalis* culture supernatant intervention was performed as mentioned above.

The intracellular ROS was measured using a 2′,7′-dichlorodihydrofluorescein-diacetate (DCFH-DA, Beyotime, Jiangsu, China) fluorescent probe according to the method described previously (Eruslanov et al., 2010). Briefly, cells were incubated with the DCFH-DA fluorescent probe (10 μM ) for 15 min at room temperature in the dark. Afterwards, the cells were washed with PBS and subsequently analyzed using a CytoFLEX Flow Cytometer (Beckman Coulter, FL, USA). Live cells were gated based on forward scatter (FSC) and side scatter (SSC) values, and DCF fluorescence was measured in the FL1 channel (488 nm excitation/525 nm emission). Data from 1× 10^4^ events at least per sample were analyzed using FlowJo™ v10 software (BD Biosciences, CA, USA).

The detection of apoptotic cells was performed by flow cytometry using the Annexin V-FITC/PI apoptosis detection kit (Solarbio Biotech Co., Ltd., Cat#CA1020, Beijing, China) according to manufacturer’s protocols. About 1 × 10^5^ cells were harvested and resuspended in 100 μL 1 × binding buffer. Then 5 µL of FITC-labeled Annexin V was added and incubated at room temperature for 15 min in the dark. Thereafter, PI (10 μL) was added and incubated for 5 min in the dark followed by addition of 400 μL of 1 × Annexin V binding buffer. Cell apoptosis analysis was detected using a CytoFLEX Flow Cytometer (Beckman Coulter, FL, USA) and analyzed with the FlowJo™ v10 software (BD Biosciences, CA, USA).

***RNA interference***

Mt2 gene knockdown was performed by siRNA interference. Specifically, the Caco-2 cells seeded at 6-well plates were then transfected with a mixture of negative control siRNA or siMt21/2/3. The mixture was formulated with RNAiMax and Opti-MEM, with a concentration of 70 nM siRNA per well. After 24 h incubation, the medium was replaced, treated with arsenic plus *Roseburia intestinalis* culture supernatant at 48 h, and harvested at 72 h after transfection. The efficiency of RNAi was analyzed using quantitative PCR. The siRNAs for clathrin heavy-chain were obtained from GenePharma (Shanghai, China), and the oligo sequences are provided in Supplementary Table S2.

***Supporting Results***

**Supplemental table S1 Primer sequences for qPCR**

| Gene | Forward Primer | Reverse Primer |
| --- | --- | --- |
| *Il-1β* | TCAACAAGATAGAAGTCAAGAG | TTGTGAGGTGCTGATGTA |
| *Tnf-α* | CATCTTCTCAAAATTCGAGTGACAA | TGGGAGTAGACAAGGTACAACCC |
| *Il-6* | CATCCAGTTGCCTTCTTG | ATTAAGCCTCCGACTTGT |
| *Il-10* | AGCAGGTGAAGAGTGATT | TTGGTGAGTAGACAGAGG |
| *Tjp-1* | CGTGGATACTTTATACAATGG | TCAGCTCTGTTCTTATTAGG |
| *Occuludin* | CTGCTGCTGATGAATATAATAG | CCTCTTGATGTGCGATAA |
| *Igfbp5* | ACCGCAAAGGATTCTACA | GCTTCATTCCGTACTTGTC |
| *Krt5* | ACATTAACAACCTCCGTAGA | TCCACATCCTTCTTCAACA |
| *Mt2* | ATGGACCCCAACTGCTCCT | TCAGGCGCAGCAGCTGCAG |
| *T-bet* | GTGGAGGTGAATGATGGA | ATCTCTGCGTTCTGGTAG |

**Supplemental table S2 siRNA sequences for RNA interference**

| siRNA | Sence Strand | Antisence strand |
| --- | --- | --- |
| *MT2-human-71* | GCAAAUGCACCUCCUGCAATT | UUGCAGGAGGUGCAUUUGCTT |
| *MT2-human-47* | CCGGCUCCUGCAAAUGCAATT | UUGCAUUUGCAGGAGCCGGTT |
| *MT2-human-13* | CCAGGGCUGCAUCUGCAAATT | UUUGCAGAUGCAGCCCUGGTT |

**
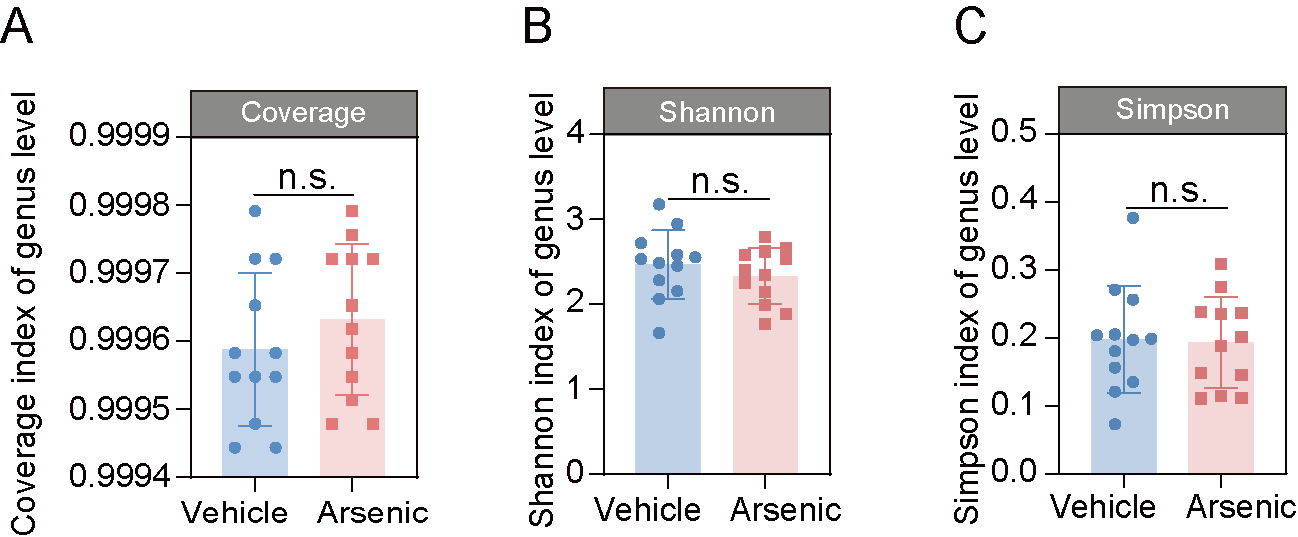
**

**Supplemental Figure S1** Alpha diversity of gut microbiota assessed by Coverage, Shannon and Simpson indexes. (A) The Coverage index. (B) The Shannon index. (C) The Simpson index. (n=12).


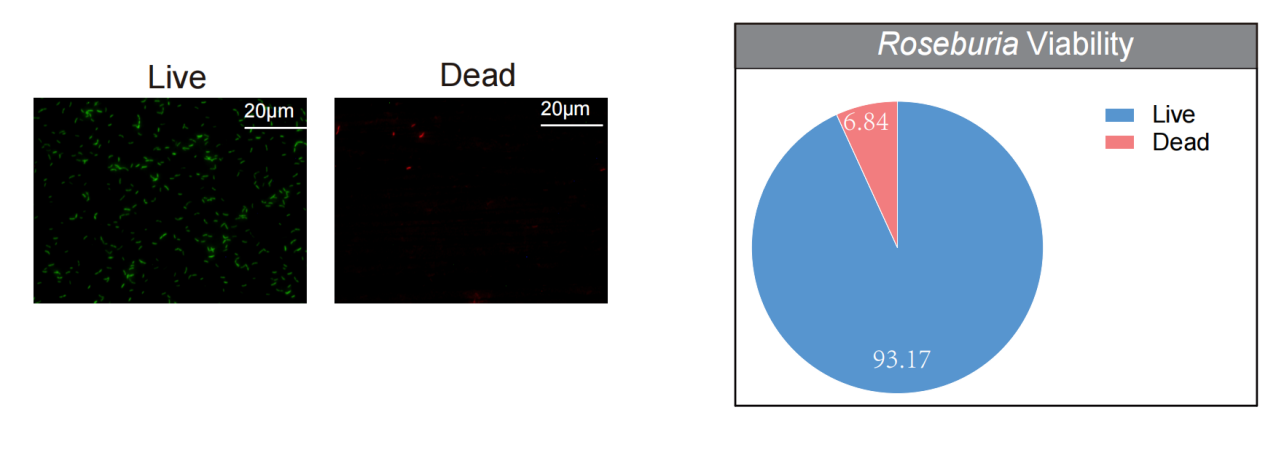


**Supplemental Figure S2** The viability of the in vitro cultivated *Roseburia intestinalis* (n=3).


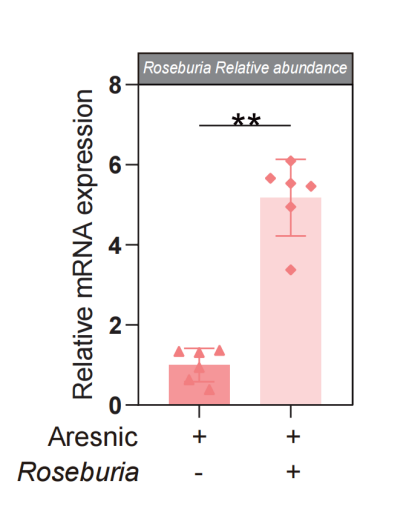


**Supplemental Figure S3** The *Roseburia intestinalis* abundance in feces of mice (n=6). * *p*<0.05; ** *p*<0.01.

**
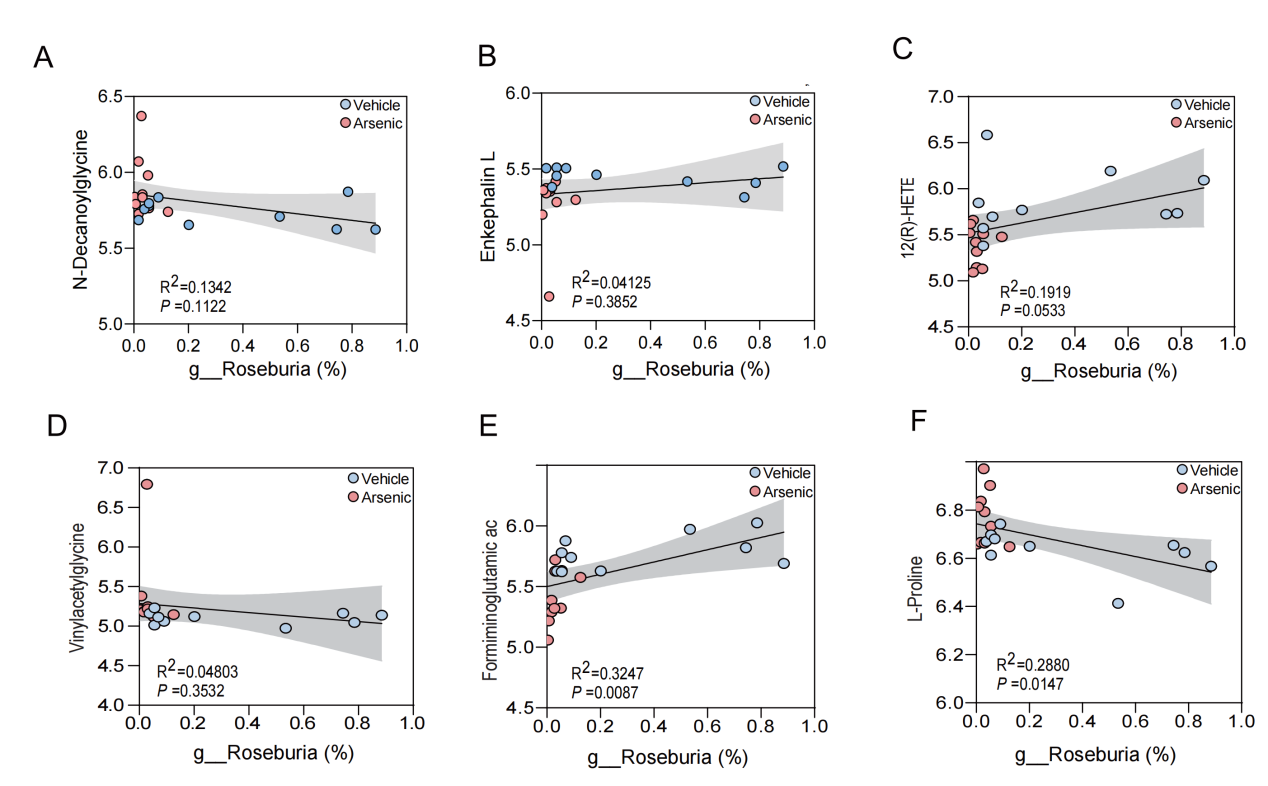
**

**Supplemental Figure S4** Spearman’s correlations between *Roseburia intestinalis* abundance and the major differently expressed metabolites. (A) The correlation between *Roseburia intestinalis* abundance and N-Decanoylglycine. (B) The correlation between *Roseburia intestinalis* abundance and Enkephalin L. (C) The correlation between *Roseburia intestinalis* abundance and 12 (R)-HETE. (D) The correlation between *Roseburia intestinalis* abundance and vinylacetylglycine. (E) The correlation between *Roseburia intestinalis* abundance and formiminoglutaminc acid. (F) The correlation between *Roseburia intestinalis* abundance and L-proline (n=10).


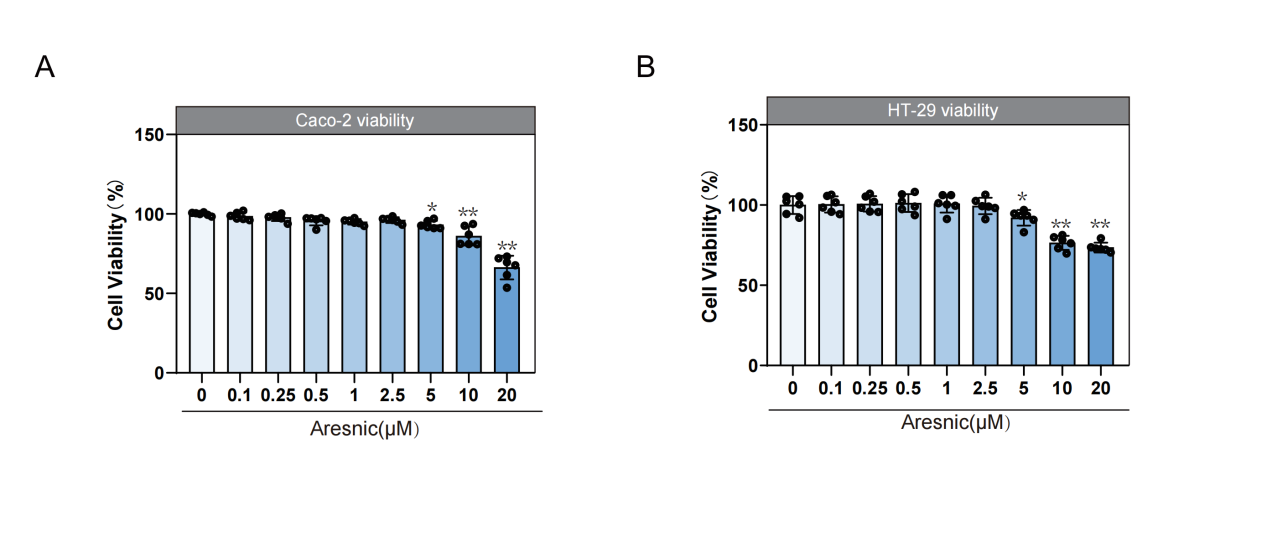


**Supplemental Figure S5** Viability of Caco-2 and HT-29 cells after exposure to graded concentrations of arsenic (n=6). * *p*<0.05; ** *p*<0.01.


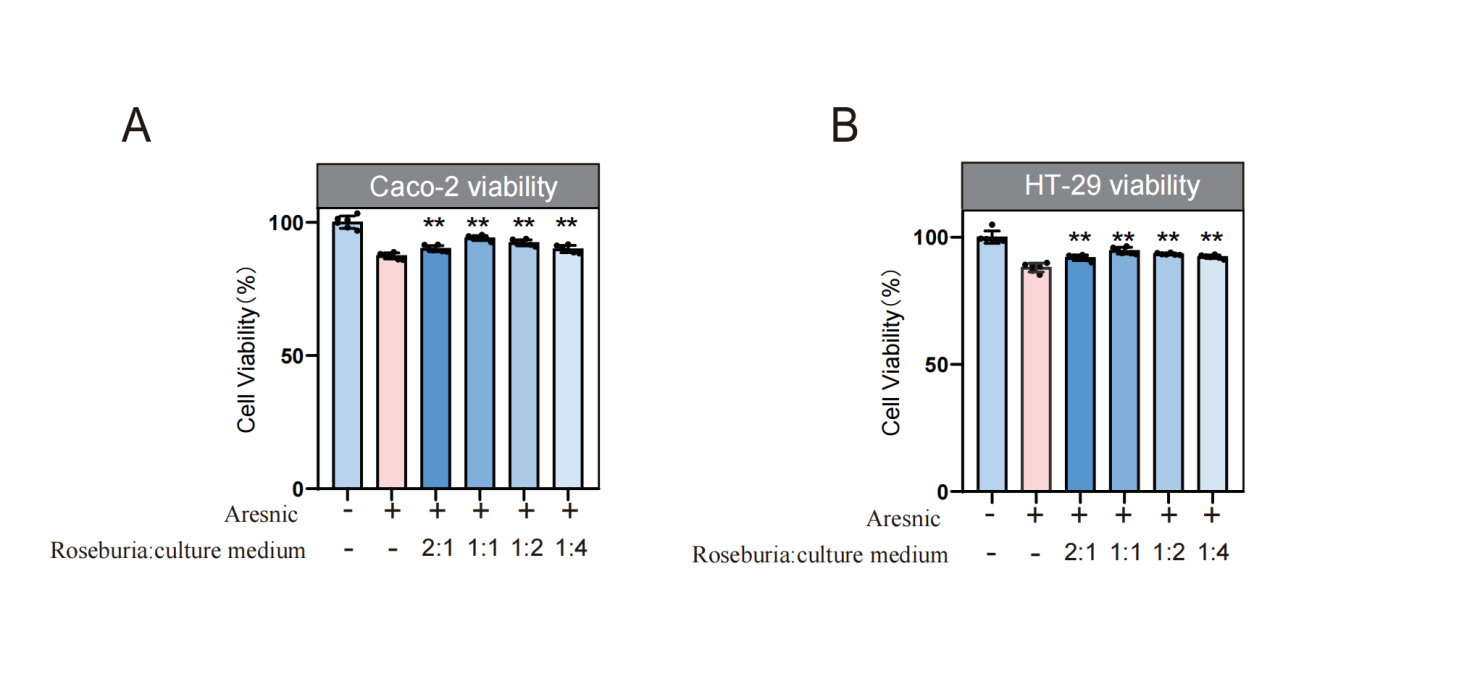


Supplemental Figure S6 The viability of Caco-2 and HT-29 cells after arsenic treatment and serially-diluted *Roseburia intestinalis* culture supernatant intervention. (A) The viability of Caco-2 cells after arsenic treatment and serially-diluted *Roseburia intestinalis* culture supernatant intervention. (B) The viability of HT-29 cells after arsenic treatment and serially-diluted *Roseburia intestinalis* culture supernatant intervention (n=6). ** *p*<0.01.


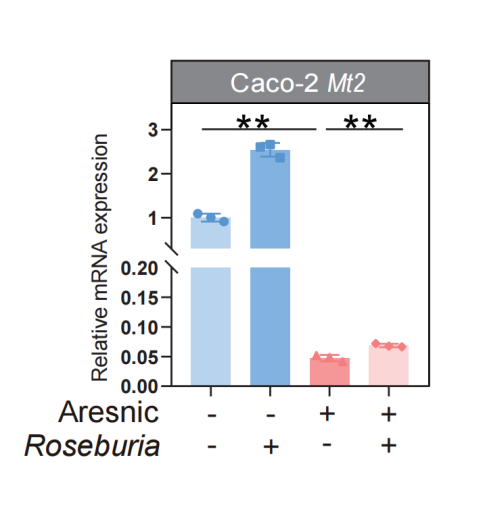


Supplemental Figure S7 *Mt2* expression in Caco-2 cells under arsenic treatment with or without the *Roseburia intestinalis* culture supernatant intervention (n=3). ** *p*<0.01.


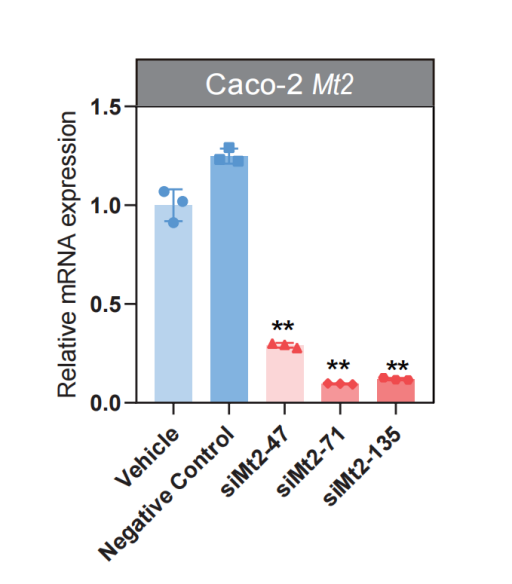


Supplemental Figure S8 *Mt2* expression after different siRNAs transfection in Caco-2 cells (n=3). ** *p*<0.01.


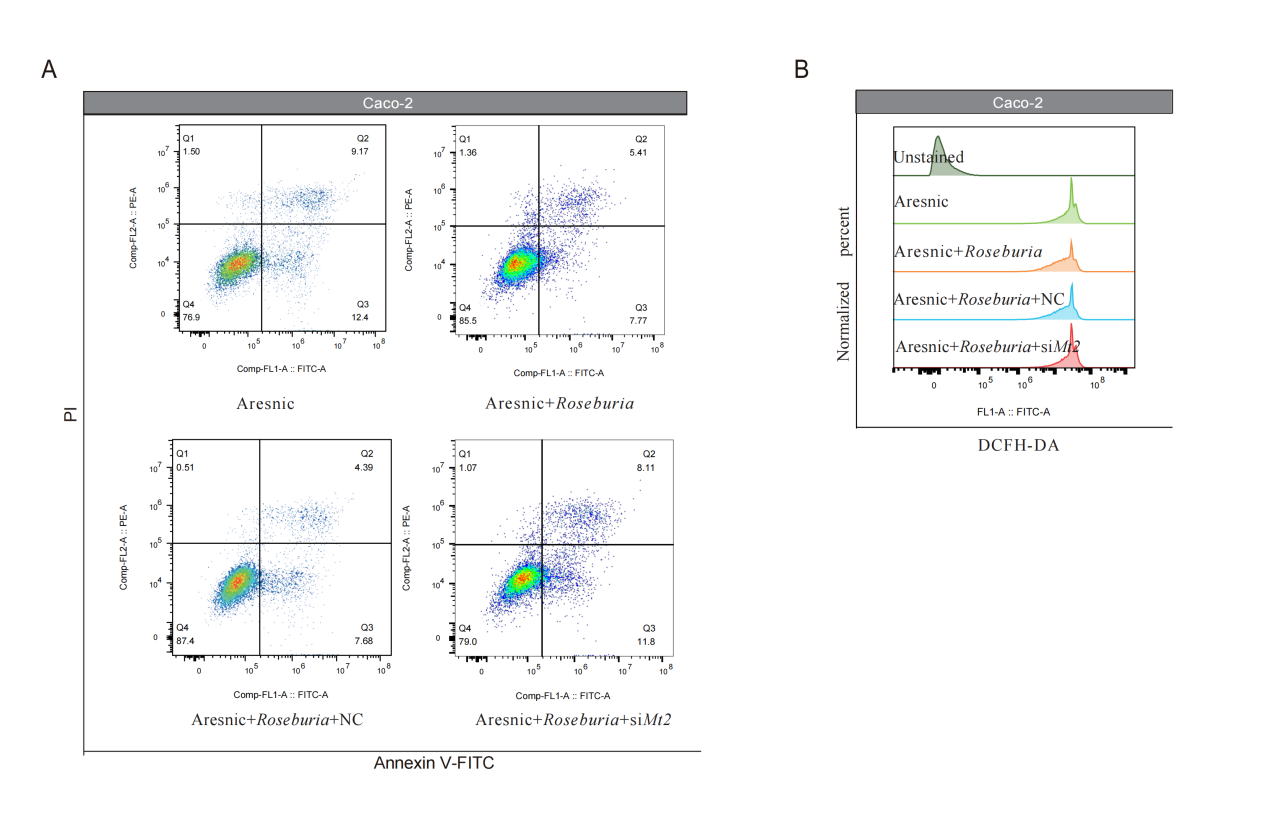


Supplemental Figure S9 The representative flow cytometry analysis of apoptosis and intracellular ROS in Caco-2 cells treated with arsenic plus *Roseburia intestinalis* culture supernatant intervention, with or without *Mt2* knockdown. (A) The representative flow cytometry analysis of apoptosis in Caco-2 cells treated with arsenic plus *Roseburia intestinalis* culture supernatant intervention, with or without *Mt2* knockdown. (B) The representative flow cytometry analysis of intracellular ROS in Caco-2 cells treated with arsenic plus *Roseburia intestinalis* culture supernatant intervention, with or without *Mt2* knockdown.
